# The induction of the fibroblast extracellular senescence metabolome is a dynamic process

**DOI:** 10.1038/s41598-018-29809-5

**Published:** 2018-08-14

**Authors:** Emma N. L. James, Mark H. Bennett, E. Kenneth Parkinson

**Affiliations:** 1 Centre for Immunobiology and Regenerative Medicine, Institute of Dentistry, Barts and the London School of Medicine and Dentistry, Queen Mary University of London, Turner Street, London, E1 2AD UK; 2 Department of Life Science, South Kensington Campus, Imperial College London, London, SW7 2AZ UK

## Abstract

Cellular senescence is often associated with irreparable DNA double strand breaks (IrrDSBs) which accumulate with chronological age (IrrDSBsen). The removal of senescent cells ameliorates several age-related diseases in mice but the translation of these findings into a clinical setting would be aided by the characterisation of non-invasive biomarkers of senescent cells. Several serum metabolites are independent indicators of chronological age and some of these accumulate outside senescent fibroblasts independently of cell cycle arrest, repairable DNA breaks and cell size (the extracellular senescence metabolome, or ESM). The post-mitotic phase of senescence is dynamic, making the detection of senescent cells *in vivo* difficult. An unbiased metabolomic screen of the IrrDSBsen fibroblast ESM also showed differences in the times of initiation and maintenance of different metabolites but generally the ESM altered progressively over the 20 day study period unlike the reported transcriptional profiles. This more detailed analysis of IrrDSBsen identified several new ESM metabolites that are associated with chronological ageing. Targeted analysis of citrate confirmed the dynamic nature of this metabolite in two cell lines and revealed its independence from the senescence effector p16^INK4A^. These data will aid our understanding of metabolic signatures of ageing and their relationship to cellular senescence and IrrDSBs.

## Introduction

The continuous culturing of human cells until they stop proliferating after a variable number of divisions is known as replicative senescence or proliferative exhaustion (PEsen) and is countered by telomerase; an enzyme that maintains the telomere repeats at the ends of chromosomes. The senescent growth arrest, which is irreversible under normal circumstances, is dependent on the p16^INK4A^/pRb and p53/p14^ARF^ tumour suppressor pathways^1^ and can be triggered in telomerase-deficient dividing cells to induce mitochondrial damage^2^ which then amplifies the phenotype by, amongst other things, inducing reactive oxygen species^3^. PEsen operates in some cell types *in vivo*, when telomeres are rendered dysfunctional, to induce irreparable DNA double strand breaks (IrrDSBs) and a DNA damage response^4^. In addition, whereas most DNA double strand breaks are readily repaired, telomeres can accumulate IrrDSBs in non-dividing tissues such as the brain and liver^5,6^, owing to their inability to repair double strand breaks by non-homologous end joining^5^. Therefore, IrrDSBs at telomeres can contribute to senescence and ageing in both dividing and non-dividing tissues.

In addition to becoming growth arrested, senescent cells also secrete an array of molecules known as the senescence-associated secretory phenotype (SASP) that consists of cytokines, growth factors and tissue remodelling enzymes^7^. While these factors are part of the important role played by senescent cells in wound healing^8,9^, if present in the wrong context they can cause inflammation and damage to surrounding cells (reviewed in^10^. Some of the SASP factors have been shown to spread the senescent phenotype both *in vitro* and *in vivo*^11^ and so a small number of senescent cells within a tissue can cause more widespread damage. Furthermore, in diseases such as oral fibrosis the number of fibroblasts expressing markers of IrrDSBs is much higher (13.6%) than either p16^INK4A^ (7.3%), senescence-associated beta galactosidase (1.4%) or a marker of senescence-associated heterochromatic foci (2.1%)^9^ and as the SASP is induced relatively early in the induction of IrrDSBsen^12^, cells harbouring IrrDSBs could have an impact on tissue function.

The process of ageing has long been known to be associated with many human diseases but until recently the role of senescent cells in this process was somewhat controversial. However, cellular senescence has now been identified in numerous human pathologies associated with ageing and the genetic or pharmacological deletion of certain types of senescent cells in mouse models delays or reverses many age-related diseases, including atherosclerosis, kidney dysfunction, osteoarthritis osteoporosis and cancer^13^, in addition to the side effects of conventional cancer therapies^14^.

It is now thought that cellular senescence may have evolved as an alternative to apoptosis to clear damaged cells in conjunction with the innate and adaptive immune systems^15^, or to participate in tissue remodelling during wound repair^16^, development^17,18^ and fibrosis^19^. Despite clear evidence of a beneficial role for senescent cells in these processes, overall their transient deletion does not significantly affect wound repair or fibrosis^20^. Thus there is considerable interest in the development of drugs that specifically sensitise senescent cells to apoptosis or otherwise delete senescent cells (senolytics). Some of these appear to work in mice^21^ but have unacceptable side effects in humans and so the search for new senolytics is a current active area of research^13^. Other approaches include boosting the immune system, targeting the SASP^13^ and reversing or delaying senescence. However, if any anti-senescence therapy is to work, including dietary supplements, biomarkers of their action in the pathologies where senescent cells are suspected to be involved will be required to monitor their effectiveness, and the molecular and cellular basis of these markers will need to be understood.

We have characterised the extracellular metabolome of several human fibroblast lines induced to senesce by either proliferative exhaustion (PEsen) or irreparable DNA double strand breaks (IrrDSBsen) that are independent of cell cycle arrest (the extracellular senescence metabolome or ESM)^22^. Several of these metabolites have been shown to be independent indicators of chronological age in humans adjusting for other parameters such as telomere length, sex, BMI, batch effect and family relatedness. In addition, they are also independent of age-related parameters such as cholesterol, albumin and low birth weight^23^.

One ESM metabolite, extracellular citrate (EC), has been followed up in more detail using targeted mass spectrometry analysis and the following points have been established. Citrate is depleted within the cell but accumulates between 2 and 11 fold externally, showing that the mechanism of its accumulation is not merely a consequence of increased biomass in senescent cells^22^. This assertion has now been confirmed directly as several manipulations that regulate EC do not affect cell size and vice versa; EC is also tightly associated with senescence and irreparable DSBs (James *et al*. - manuscript in preparation). Interestingly, serum citrate does not accumulate in a linear fashion with chronological age in humans but increases over the age of fifty to sixty-five years of age when age-related diseases begin to rise^24^. Blood citrate levels are also strikingly elevated in age-related diseases such as non-alcoholic fatty acid disease^25^ and type 2 diabetes^26^ where senescent cells are known to accumulate^27^ and to be instrumental in the development of the diseases^28^.

It has long been known that the post-mitotic phase of cellular senescence is a dynamic process, with high levels of p53 and p21^WAF^ giving way to lower levels of p53/p21^WAF^ and accumulating p16^INK4A^ ^29,30^. Recently, detailed bioinformatics and single cell transcriptomic analyses of IrrDSBsen has confirmed and extended these earlier observations to show considerable temporal variations and heterogeneity in the senescent cell transcriptome when senescence is induced by a variety of stimuli, all of which are reported to involve the generation of irreparable DNA double strand breaks^31,32^. However, transcriptomics is not readily applicable to the non-invasive monitoring of anti-senescence therapies and as most age-related diseases are polygenic genome-wide association studies have, as yet, explained only a small fraction of these diseases; they have also only made only a small contribution to understanding disease –related mechanisms^33^. As metabolomics potentially gives an integrated readout of transcriptomics and proteomics and is suited to non-invasive technology^33^, it presents an opportunity to identify and characterise metabolites that are associated with irreparable DNA double strand breaks and/or senescence. However, it is important to understand the basis for age-associated metabolites and in particular their relationship to IrrDSBs and senescence. It is also important to identify metabolites (and other non-invasive biomarkers) of forms of senescence that are independent of p16^INK4A^, as p16^INK4A^ is not induced in some cell types following senescence^31^ and is transcriptionally silenced in 47% of histologically normal mammary epithelial tissues *in vivo*^34^. Senescence in the absence of p16^INK4A^ expression or upregualtion has been reported following telomere attrition and/or the upregulation of p21^WAF/CIP1^ in human mammary epithelial cells^35^ keratinocytes^36^ and fibroblasts^37,38^.

As the transcriptome of senescent cells is dynamic, we hypothesised that same was true for the IrrDSBsen ESM. We therefore re-examined several of our other unbiased metabolomics screens of the IrrDSBsen where we had conducted time courses following a doses of irreparable and repairable ionising radiation-induced DSBs and compared the data with that of PEsen. The results show that the ESM, like the transcriptome, is dynamic. Some metabolites alter early; more closely associated with the acquisition of DSBs than the mature senescent phenotype where the cells become p16^INK4A^-positive. However, unlike the recently published transcriptional profiles of IrrDSBsen induction^31^ most profiles continue to alter progressively following the induction of IrrDSBs and very few alter and then return to normal.

We also tested the hypothesis that some metabolites associated with human chronological ageing^23^ might be altered early in the induction of IrrDSBsen but not at later time points. This might mean that early metabolites would be absent from the PEsen and IrrDSBsen ESMs published previously^22^ but with the exception of asparagine and glutamate, we did not find that this was the case. Many of the IrrDSBsen metabolites were not reported in the PEsen screen because they were also elevated in the quiescence controls and were thus not specific markers of senescence^22^ although they were reported to be associated with chronological ageing *in vivo*^23^. However, we did identify some new metabolites that met the criteria of senescent cell specificity.

In addition we confirmed that the accumulation of EC in two cell lines was also dynamic in nature and showed that it accumulated modestly following the establishment of IrrDSBs but continued to accumulate as senescence became established. These data also showed that p16^INK4A^ was unnecessary for EC accumulation following IrDSBsen.

## Results

### The IrrDSBsen ESM is a dynamic process

A recent study of the dynamics of the induction of IrrDSBsen showed that the transcriptome varies dramatically of the senescence programme from when it is first induced to its establishment at day 20^31^. We have previously characterised the extracellular metabolites of human oral fibroblasts induced to senesce by proliferative exhaustion (PEsen). We applied very strict criteria when producing the list of metabolites that we termed the ‘extracellular senescence metabolome’ (ESM). The metabolite had to be elevated compared to growing cells in more than one PEsen line but not elevated in quiescent cells induced by serum-starvation or confluence, and elevated in more than one line induced to senesce by IrrDSBs but not elevated in cells with repairable levels of DNA damage^22^. However, we have not previously analysed the dynamics of IrrDSBsen initiation and establishment to gain an insight into whether the metabolites are induced early when IrrDSBs form, late when senescence becomes established, or both. An unbiased metabolomics screen identified 190 extracellular metabolites following the induction of irreparable levels of DNA damage by 20 Gy of gamma irradiation in oral fibroblast lines NHOF-1 and NHOF-5 at 0, 5, 10 and 20 days. In this screen only 9 metabolites were statistically elevated in both cell lines independently at 20 days when compared with un-irradiated controls: 2-aminoadipate, 3-hydroxyisobutyrate caprylate, caproate, cysteine, cysteine glutathione disulphide, oxidised glutathione, pyridoxal and pyridoxate. When both NHOF-1 and NHOF-5 were combined and their zero time controls compared with their corresponding levels at day 20, 39 metabolites were significantly altered but the previously reported citrate only showed a strong trend, because of a large outlier in the NHOF-1 control group (Supplementary Table S1A). Several of these metabolites have been reported before as part of the PEsen ESM and are depicted in bold in Supplementary Table S1A ^22^. Interestingly, of the 40 metabolites listed (including citrate) 17 have been reported to be associated with chronological age in humans^23^ and 15 accumulated in the IrrDSBsen ESM with only two being depleted. Of the 23 IrrDSBsen metabolites that were not associated with chronological age *in vivo* 7 were depleted, and therefore would be expected to be inversely associated with human chronological age and of the remaining 16 only 5 were detectable in other screens of IrrDSBsen and PEsen. These results strengthen the argument that the ESM overlaps with the serum metabolites associated with chronological ageing.

**Figure 1:**
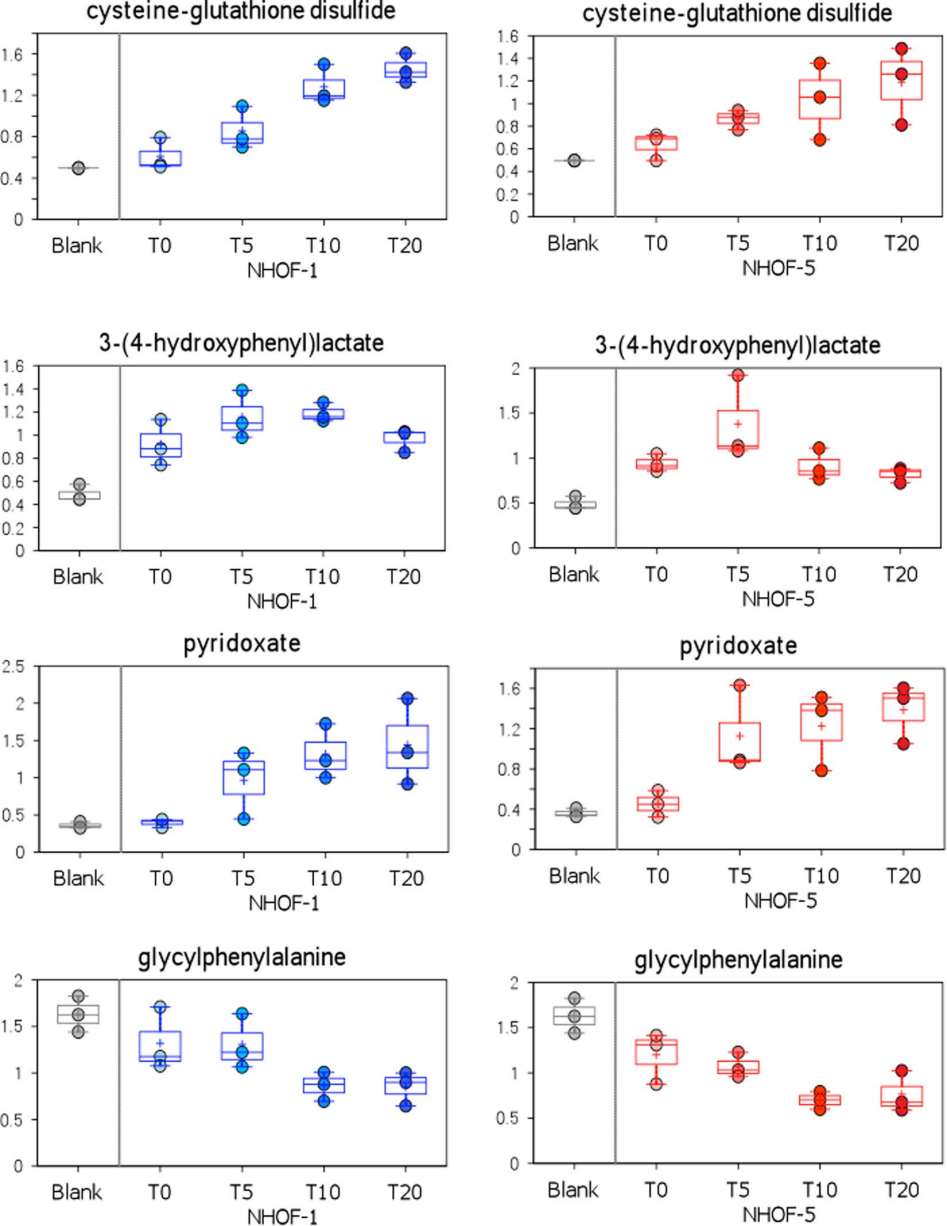
Examples of the time course of PEsen ESM metabolites following the induction of IrrDSBsen. The figure shows examples of four different patterns of ESM kinetics. Late accumulation (cysteine glutathione disulphide), early accumulation followed by late decline (3-(4-hydroxyphenyl)lactate), early accumulation followed by stabilisation (pyridoxate) and late depletion (glycylphenylalanne). The figure shows the time course of each detectable ESM PEsen metabolite after the induction of IrrDSBsen over a 20 day period in two oral fibroblast lines NHOF-1 (blue) and NHOF-5 (red). The symbols for the box and whisker plots are as follows; + = mean value, the horizontal lines within the boxes = median value, O = extreme points, the limits of the boxes = the upper and lower quartiles and the error bars indicate the maxima and minima of distribution.

We then examined whether some metabolites were statistically significantly altered at day 5 or day 10 if the results of both cell lines were combined, but not at day 20 following ionising radiation (Fig. 1 and Supplementary Table S1B). We found that asparagine, 2-hydroxybutyrate and 3-(4-hydroxyphenyl)lactate were elevated at day 5 only to decline by 10 and 20 but only in the case of 3-(4-hydroxyphenyl)lactate was this obvious in both cell lines studied. However, all three of these early onset ESM changes have been reported to be associated with human ageing^23^ and might have been missed by our earlier PEsen ESM analysis. When we examined the detectable PEsen ESM metabolites (Fig. 1, Supplementary Fig. S1), we noticed that most were altered at day 5, in keeping with the establishment of IrrDSBs and the induction of the SASP^12^. These same metabolites then gradually changed further, only stabilising between 10 and 20 days after DSB induction (e.g. dihomo-linoleate (20:2n6), all dipeptides, gamma-glutamylmethionine cysteine glutathione disulphide and many other metabolites (Fig. 1)). However, some accumulated significantly only at day 20 (e.g. citrate, urate and erythronate). Yet more metabolites were altered at day 5 and were sustained for the entire 20 day experiment (e.g. pyridoxate, 1-stearoylphosphoglyceroinositol). None of the metabolites detected in the PEsen screen published previously^22^ were elevated at day 5 and then declined by day 10 and day 20 (Supplementary Table S1B) and so some metabolites altering early might have been missed by the PEsen screen. The results are not necessarily related to the well-known increase in senescent cell biomass as several ESM metabolites are depleted inside the cells whilst accumulating outside (e.g. citrate – see ref.^22^) and as can be seen from Supplementary Fig. S1 some metabolite alterations are not even detectable above background at T0 (e.g. urate, oxidised glutathione, pyridoxate).

**Figure 2:**
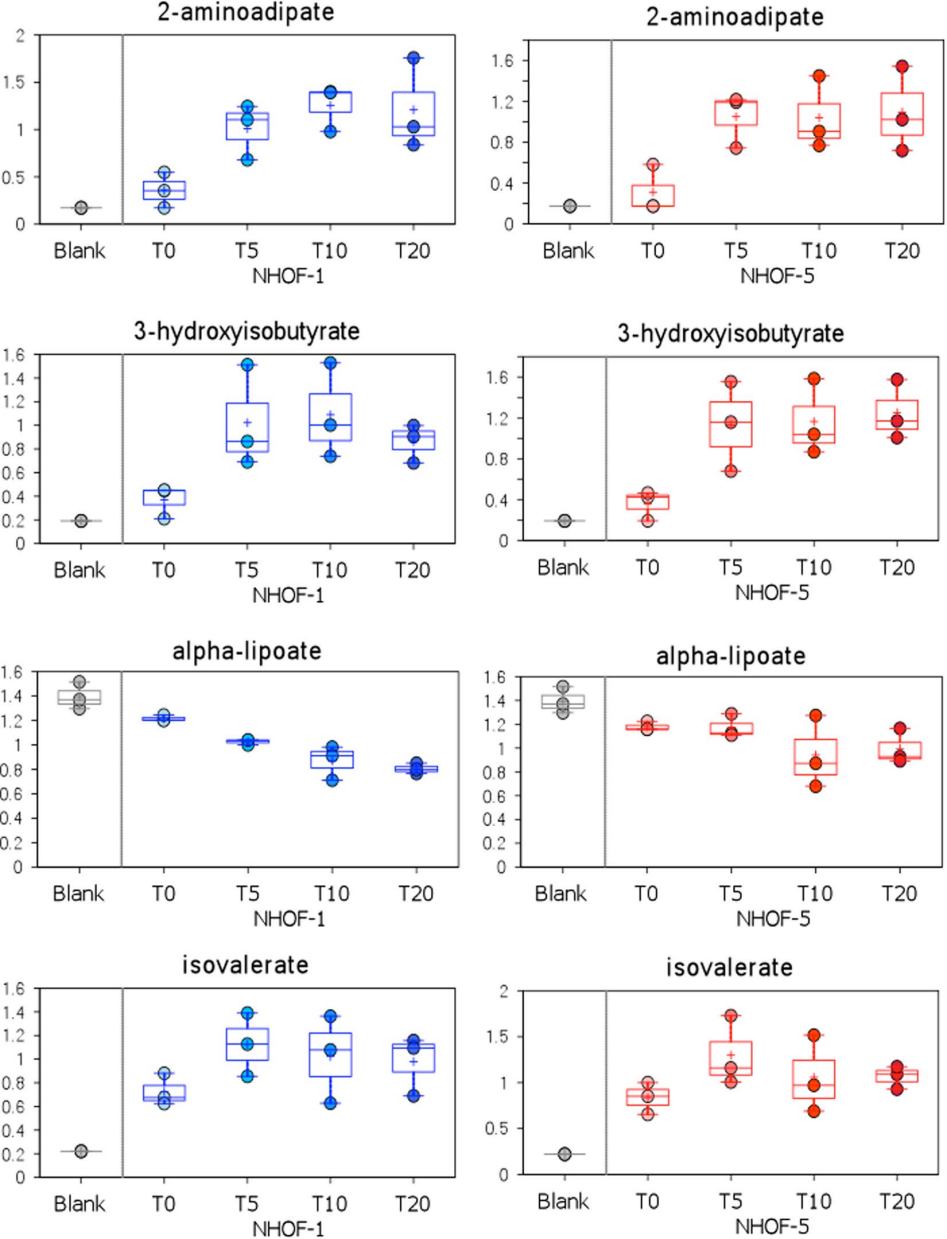
Examples of the time course of novel ESM metabolites following the induction of IrrDSBsen. The figure shows the IrrDSBsen ESM kinetics for four IrrDSBsen metabolites not previously identified in the PEsen ESM. The symbols are the same as for Fig. 1.

In addition to asparagine, 2-hydroxybutyrate and 3-(4-hydroxyphenyl)lactate, we identified several other metabolites in the IrrDSBsen ESM that we had rejected from our PEsen ESM screen. They were 2-aminoadipate, alpha lipoate and two breakdown products of branch chain amino acid (BCAA) metabolism 3-hydroxyisobutyrate and isovalerate (Fig. 2). Caprate (10.0), caprylate (8.0) and trans-4-hydroxyproline (Supplementary Fig. S2) also accumulated in IrrDSBsen cells relative to the controls.

The main conclusion from the analysis of the IrrDSBsen time course is that unlike the transcriptome^31^, the IrrDSBsen ESM evolves gradually over time in both cell lines studied, with very few metabolites altering at day 5 or day 10 only to normalise later at day 20.

### IrrDSBsen metabolites are associated with IrrDSBs but not always specific for senescence

We went on to examine two other unbiased metabolomics screens we had conducted previously and asked, firstly, whether the metabolites of the IrrDSBsen ESM not reported in the PEsen ESM were specific to IrrDSBs rather than repairable damage. Next we asked whether these metabolites were regulated in more than one fibroblast line, and whether they were transient in nature (asparagine, 2-hydroxybutyrate, 3-(4-hydroxyphenyl)lactate), and finally why they were excluded from the PEsen ESM.

**Figure 3:**
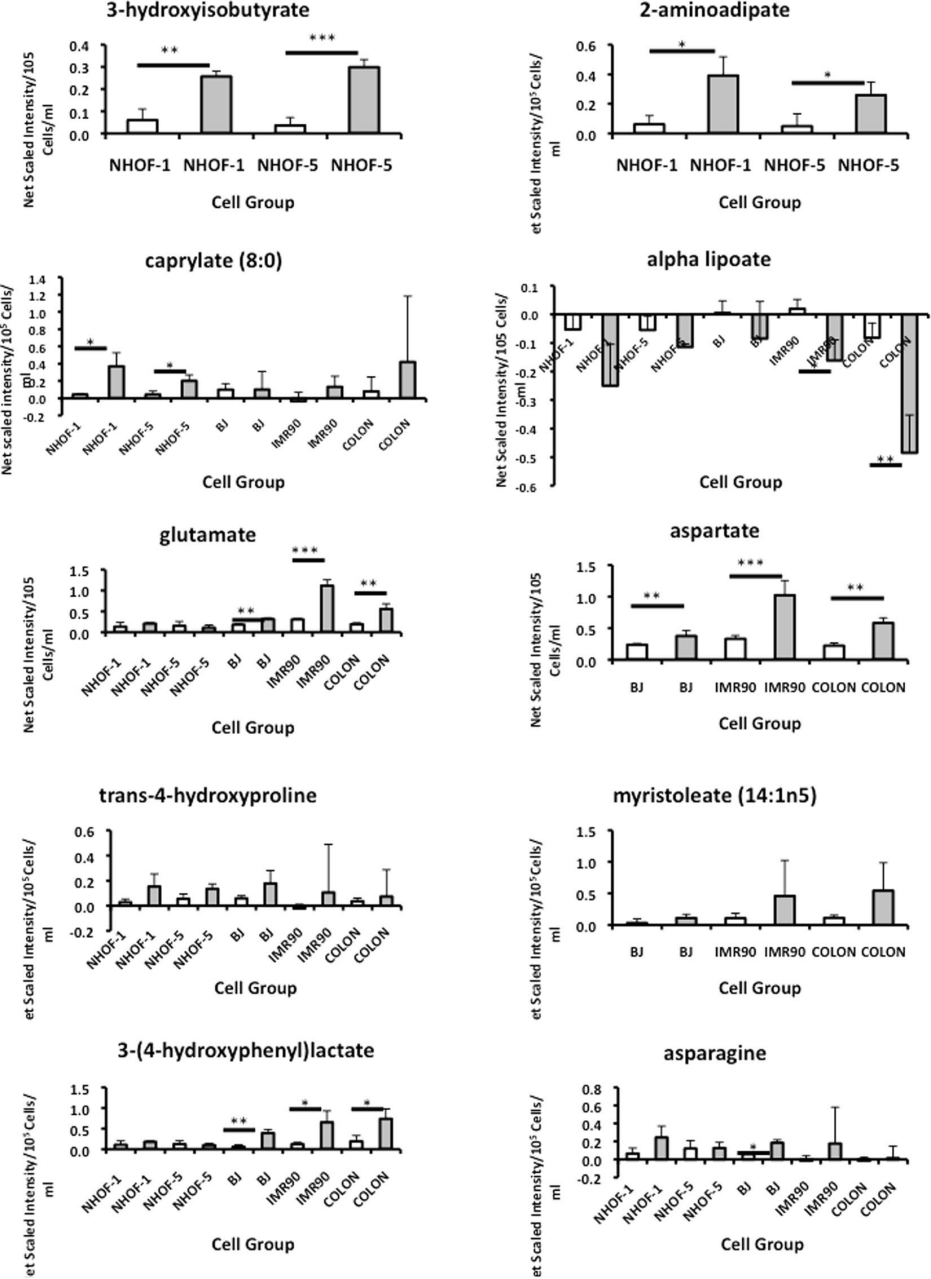
Examples of novel IrrDSBsen ESM metabolites associated with human chronological ageing. The figure shows the levels of several IrrDSBsen metabolites not detected in the previously published PEsen screen that have also been reported to be associated with human chronological ageing. The data shows relative metabolite levels following subtraction of the unconditioned medium blanks. The data shows metabolite levels at day 20 following 20 Gy of ionizing radiation relative to the growing controls in five different fibroblast lines. Dark grey bars = 20 Gy IrrDSBsen cells at 20 days; white bars = growing controls. n = 3 per group *p < 0.05 **p < 0.01 ***p < 0.001 as assessed by the two-tailed Student’s T Test. P values for 20 Gy versus growing cells for all cell lines combined are given in Supplementary Table S1.

**Figure 4:**
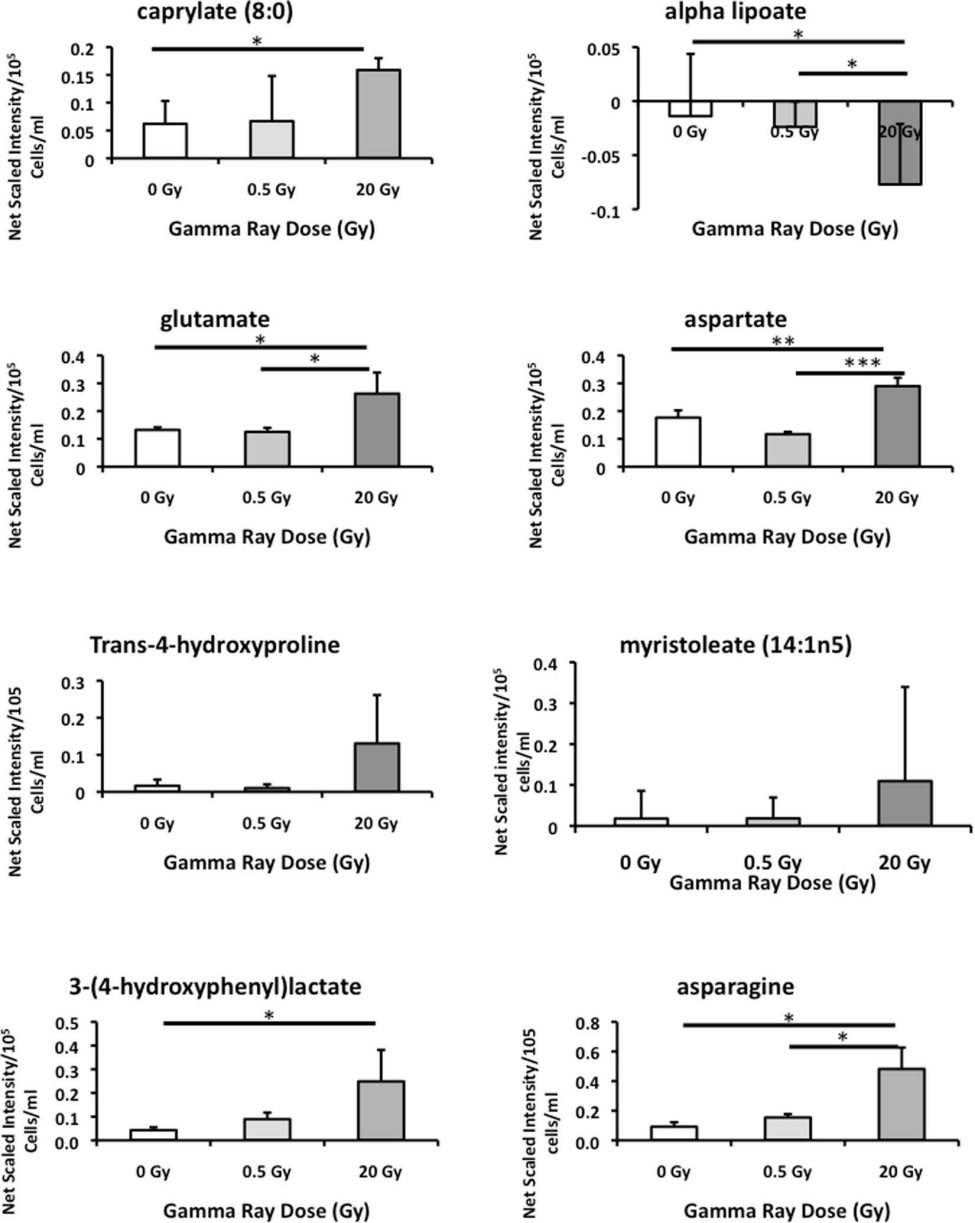
Novel IrrDSBsen ESM metabolites associated with human chronological ageing at day 5 are not induced by repairable levels of DNA damage. The figure shows the levels of the same IrrDSBsen metabolites as in Fig. 3. The figure represents relative metabolite levels following subtraction of the unconditioned medium blanks and shows the NHOF-1 extracellular metabolite levels at day 5 following 20 Gy of ionizing radiation relative to the growing controls and cells receiving a repairable dose (0.5 Gy) of ionizing radiation. Dark grey bars = 20 Gy cells; light grey bars = 0.5 Gy cells; white bars = growing controls. n = 3 per group *p < 0.05 **p < 0.01 ***p < 0.001 as assessed by the two-tailed Student’s T Test.

Figure 3 shows the results of this analysis for the novel IrrDSBsen metabolites as well as glutamate, aspartate and myristoleate (14:1n5) as these last metabolites are part of the chronological ageing signature defined by others^23^. Supplementary Table S2 shows that many metabolites were significantly increased when IrrDSBsen were compared with unirradiated controls in two independent unbiased screens and the significance increased when the results of both screens were combined. 2-hydroxybutyrate and 3-(4-hydroxyphenyl)lactate) differed much more significantly at Day 20 following irradiation when the power of the study was increased to five lines but asparagine was still not significantly different from the controls at day 20. We then went on to examine data when NHOF-1 fibroblasts irradiated with 20 Gy were compared at Day 5 with both growing controls and cells irradiated with 0.5 Gy which is repairable^12^ and allowed the cells to begin growing again (Fig. 4). Unfortunately, no data was available for 3-hydroxyisobutyrate or 2-aminoadipate as they were not detected in this particular screen but asparagine, isovalerate, aspartate and glutamate were statistically distinguishable from growing and 0.5 Gy controls at day 5. Next we went back to our PEsen screen and asked whether the IrrDSBsen metabolites were altered in PEsen NHOF-1 cells when compared to confluent quiescent controls as these cells had been identified by cluster analysis to most closely resemble PEsen cells in previous work^39^. Unfortunately, no data was available for aspartate or myristoleate (14:1n5) as they were not detected in the PEsen screen published previously but we found that 3-hydroxyisobutyrate differed statistically from all 3 controls groups, trans-4-hydroxyproline differed from the confluent group and alpha lipoate, isovalerate, caprylate (8.0) and glutamate showed a strong trend for a difference between the PEsen and confluent groups. However, asparagine was depleted compared to the confluent controls rather than accumulating, in keeping with its early transient alteration and its relationship to the establishment of IrrDSBs. In summary, whilst the ten new IrrDSBsen metabolites in Supplementary Table S2 are significantly different from growing cells only 3-hydroxyisobutyrate is specific to senescence with some others showing a trend. However, glutamate and asparagine may have been missed in the PEsen screen of fully senescent fibroblasts because they are higher at day 5 in NHOF-1 than at day 20 in some experiments.

### The IrrDSBsen ESM and markers of chronological ageing/age-related diseases overlap

We have previously shown that a quarter of the serum metabolites that are strongly associated with chronological ageing and age-related diseases independently of age^23^ are also part of the fibroblast extracellular senescence metabolome (ESM)^22^ (see also Supplementary Table S3) as induced by proliferative exhaustion (PEsen) and irreparable DNA double strand breaks (IrrDSBsen) in more than one cell line. IrrDSBsen may be more sensitive because it is more synchronously induced and can be defined over time. Furthermore, IrrDSBs have been shown to accumulate with chronological age in several post-mitotic mouse tissues^5,6^. We now report that aspartate, glutamate and the long chain fatty acids myristoleate (14:1n5), are more convincingly associated with the IrrDSBsen ESM than with the PEsen ESM published previously^22^. All were elevated in more than one cell line (Fig. 3) but only aspartate (3/4 lines) and glutamate (3/5 lines) were statistically significant at 20 days following radiation. Furthermore, both aspartate and glutamate were associated with irreparable DSBs 5 days after irradiation, as assessed by their return to normal after irradiation with repairable doses of gamma rays (0.5 Gy – see Fig. 4), whilst aspartate accumulated early (Fig. 4), was detectable in more than one cell line (Fig. 3) and was sustained for 20 days (Fig. 3). Many of the metabolites associated with human chronological age that were not associated with the ESM would not be expected to be modified *in vitro* because they are either xenobiotics (erythritol) thought be due to muscle breakdown (creatine and creatinine) or present at very high levels already in the culture medium (serine). Unfortunately no new data was available for C-glycosyltryptophan and eicosapentaenoate (EPA; 20:5n3) as these metabolites were not detected in these IrrDSBsen screens. Nevertheless, 7 of the 15 detectable metabolites reported to be independent indicators of chronological age^23^ were also elevated in our PEsen and IrrDSBsen ESM screens, although some metabolites such as citrate and urate were much more reproducible than others, and many metabolites elevated in the PEsen and IrrDSBsen ESMs did not associate with human chronological age. However, our data also suggests that whilst aspartate, glutamate and the long chain fatty acids myristoleate (14:1n5) are good independent biomarkers of chronological age^23^ and part of the ESM, they may be markers of cell cycle arrest or quiescence rather than specific markers of senescence.

### Extracellular citrate (EC) is detectable in PEsen and IrrDSBsen by targeted metabolomics and is induced independently of p16^INK4A^

One of the most reproducible and specific metabolomics markers of senescence and IrrDSBs is EC. EC has been shown to accumulate in 3 independent unbiased screens (this study and see ref.^22^) and a total of 8 different fibroblast lines undergoing IrrDSBsen (James and Parkinson – unpublished data). We therefore tested whether it was detectable by targeted metabolomics in the oral fibroblast lines NHOF-1 and IMR90 following the induction of IrrDSBs and PEsen and whether it followed the same temporal pattern of induction as in NHOF-1 and NHOF-5 in the unbiased non-targeted screen. The PEsen and IrrDSBsen cells were characterised for SA-βGal, IrrDSBs (large 53BP1 foci), Ki67 as described previously^22,39^ and also tested by western blotting for the expression of p16^INK4A^ and MCM7 established markers of late^29^ and early^40^ senescence, respectively. IrrDSBsen cells showed a marked increase in SA-βGal activity (Supplementary Fig. S3; see also ref.^31^), which peaked after 20 days in NHOF-1 (Supplementary Fig. S4) and after 10 days in IMR90 (Supplementary Fig. S3). Ki67 declined by day 5 in NHOF-1 (Supplementary Fig. S5) but by day 10 in IMR90 and to a lesser extent (Supplementary Fig. S6). However, MCM7 expression declined by day 5 in both lines (Supplementary Figs S7 and S8). IrrDSBs as assessed by large 53BP1 foci increased by day 5 in both lines and did not increase further. IrrDSBs were significantly increased in NHOF-1 at all time points (Supplementary Fig. S9) but not so in IMR90 (Supplementary Fig. S10), probably because these cells were more senescent than NHOF-1. The levels of p16^INK4A^ (Supplementary Fig. S11) increased in irradiated IMR90 by day10 and did not increase further by day 20 but NHOF-1 did not express detectable p16^INK4A^ (Supplementary Fig. S12).

**Figure 5:**
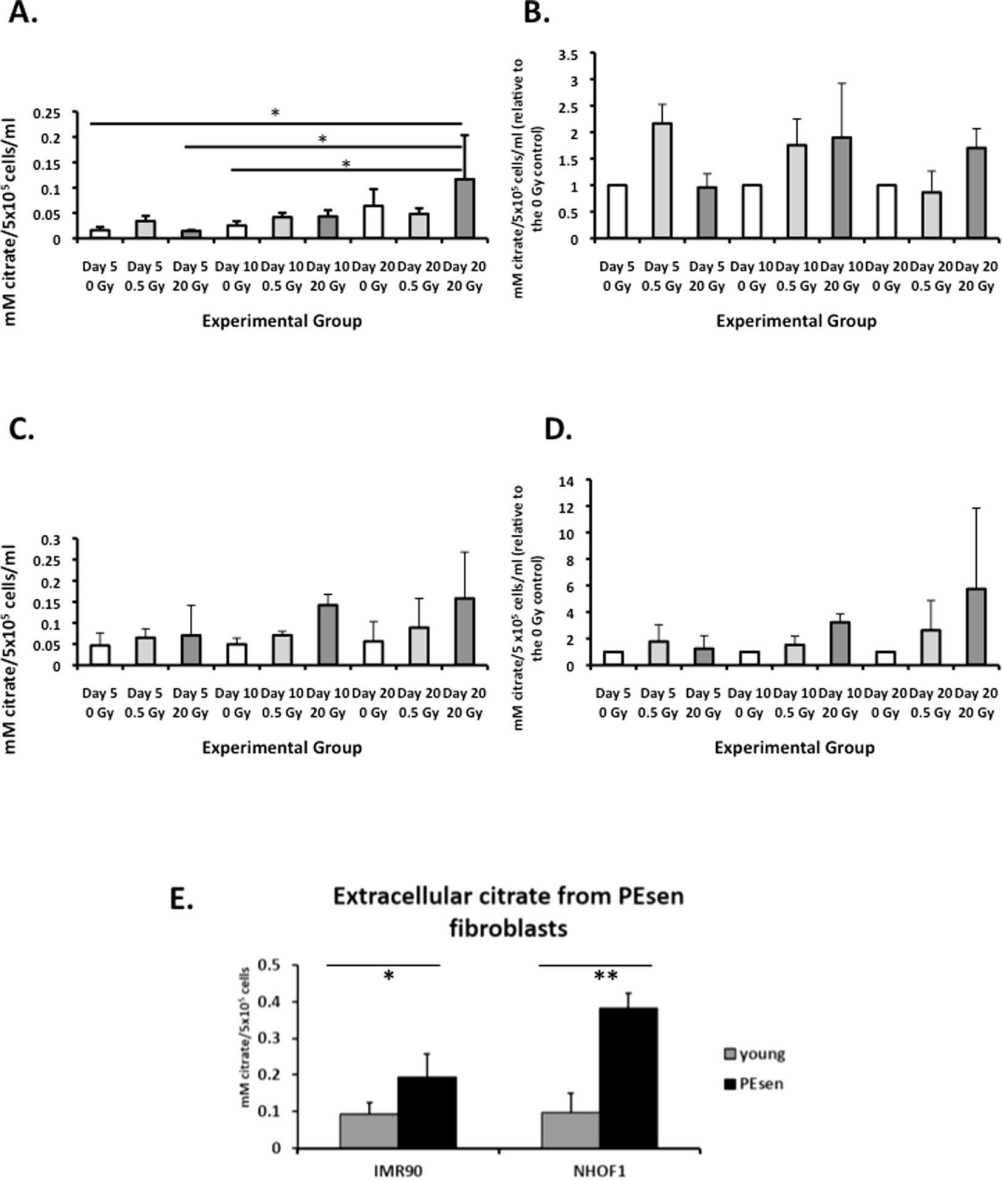
Extracellular citrate (EC) measured using GCMS. (**A** and **B**) IrrDSBsen versus controls. Extracellular citrate at 5, 10 and 20 days after irradiation with 0, 0.5 Gy and 20 Gy gamma rays. (**A**) NHOF-1; (**B**) IMR90. n = 3 except for day 10 0 Gy and 0.5 Gy in IMR90 where n = 2. Error bars represent standard deviation from the mean *p < 0.5 with a 1 way ANOVA and Tukey’s post hoc analysis. (**C** and **D**) The same data as A and B normalised to the 0 Gy control for each time point. (**E**) PEsen fibroblasts versus controls. Concentration of extracellular citrate from NHOF1 and IMR90 fibroblasts that are rendered senescent due to proliferative exhaustion (PEsen) compared to growing controls, and normalised to cell number n = 3 per group *p < 0.05 **p < 0.01 as assessed by the two-tailed Student’s T Test. Grey bars = growing controls; black bars = PEsen cells.

The levels of EC following irradiation did not increase at day 5 in NHOF-1 in this series of experiments and did not peak until day 20 at the earliest (Fig. 5A,C) but in contrast EC peaked at day 10 following irradiation in IMR90 (Fig. 5B,D). These results paralleled the induction of SA-βGal activity, which is a late and specific marker of senescence under the conditions described here. The results also show that p16^INK4A^ is not required for EC accumulation in IrrDSBsen, although the possibility that it influences the kinetics of EC induction cannot be ruled out.

PEsen NHOF-1 and the quiescence controls have been characterised previously^22,39^ but the characterisation PEsen IMR90 cells is shown in Supplementary Figs S13–S16. PEsen IMR90 showed high levels of SA-βGal activity (Supplementary Fig. S13), very little expression of MCM7 (Supplementary Fig. S14) and a large accumulation of p16^INK4A^ (Supplementary Fig. S15), which was absent in NHOF-1 (Supplementary Fig. S16). In neither NHOF-1 nor IMR90 were any of these senescence markers present in quiescent (low serum), confluent or growing controls. Targeted measurement of EC confirmed earlier reports that it was elevated in PEsen NHOF-1 and extended these observations to IMR90 (Fig. 5E). Therefore p16^INK4A^ is unnecessary for the induction of EC and other ESM metabolite accumulation in PEsen as well as IrrDSBsen

## Discussion

**Figure 6:**
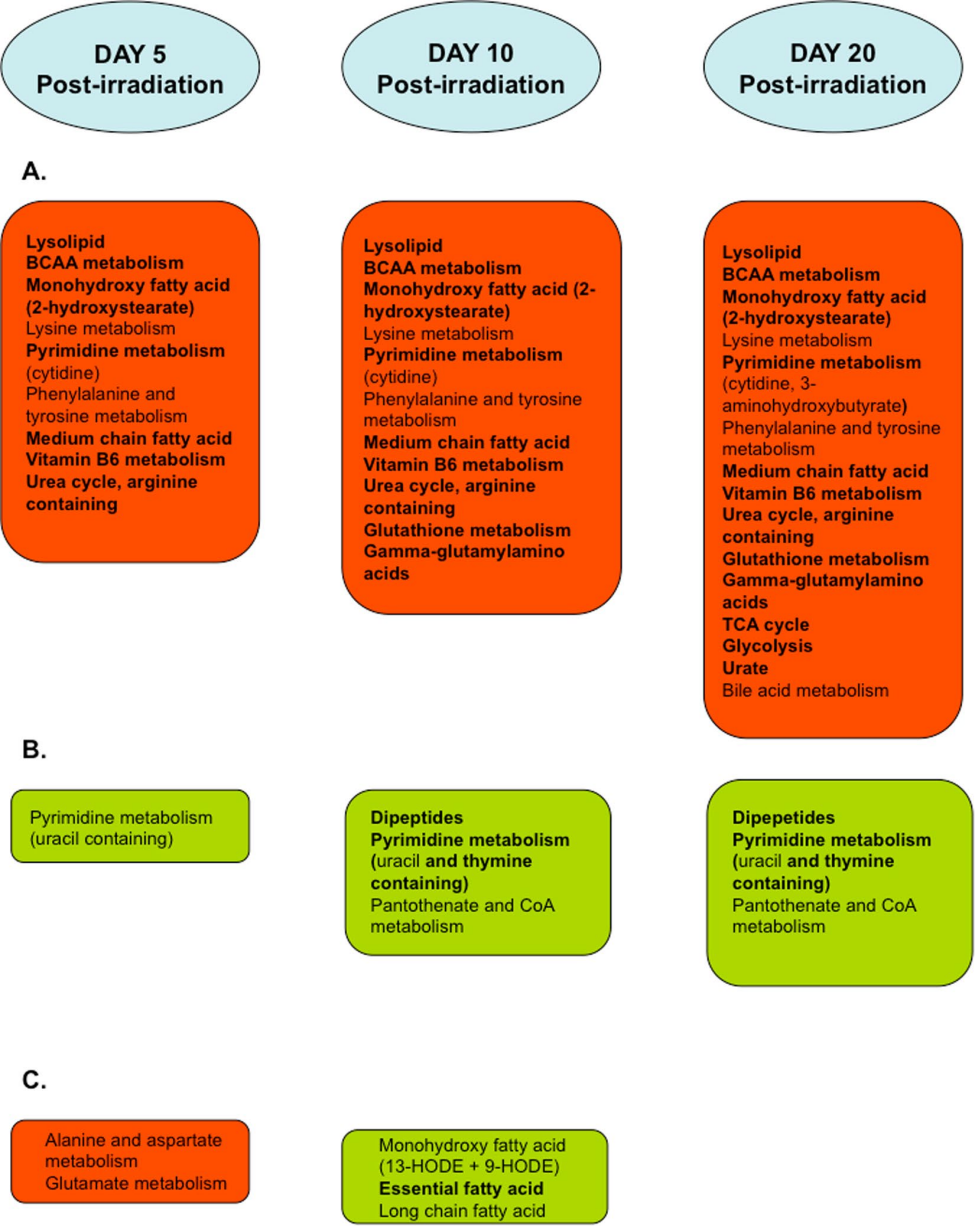
Summary of the kinetics of IrrDSBsen induction. The cartoon summarises the metabolic pathways participating in the induction and establishment of the IrrDSBsen phenotype over 20 days following 20 Gy of ionising radiation. Panels in red indicate pathways where metabolite pathways are increasing and panels in green indicate pathways where metabolites are being depleted. Pathways and metabolites appearing in bold are part of the previously reported PEsen ESM. (**A**) The pathways regulating ESM metabolites that accumulate gradually over time and are sustained with vitamin B6 metabolites such as pyridoxate accumulating early, glutathione and gamma-glutamyl amino acids later and citrate and urate later still. (**B**) The same as (**A**) but showing ESM metabolites that become depleted. The dipeptides appear to be depleted at day 10 and then remain depleted at day 10. (**C**) The pathways regulating metabolites that are transient in nature.

We have previously characterised the specific extracellular metabolites of senescent human fibroblasts, termed collectively as the extracellular senescence metabolome or ESM^22,39^. However, as recent reports have reinforced previous data to show that the post-mitotic senescent phenotype is highly dynamic and heterogeneous^31,32^, we hypothesised that our previous list of ESM metabolites, which were largely based on PEsen, might have missed key metabolites that arose early and then declined or conversely metabolites that peaked later than the assay time point. We show here that indeed the IrrDSBsen ESM is dynamic and a summary of the changes that take place over time following the induction of IrrDSBsen is given in Fig. 6. Unlike the recently published transcriptomic analysis of IrrDSBsen^31^, most metabolites began to alter at day 5 following irradiation, stabilised at day 10 and remained the same at day 20, although the timing did vary from experiment to experiment. A few metabolites did reach their peak of alteration at day 5 and remained high whilst even fewer peaked at day 5 only to decline by day 20. Virtually no metabolites began to rise at day 20, although citrate, urate, glycerate and bile acid metabolites all were higher at day 20 than day10. The results show that the ESM is more stable than the recently reported IrrDSBsen transcriptome^31^, at least under the conditions we have used here, and PEsen and IrrDSBsen ESM metabolites are specific to senescence as opposed to quiescence^22^. However, we cannot address cellular heterogeneity as the technology for single cell metabolomics is still in its developmental stage^41^, nor can we claim that the IrrDSBsen and PEsen ESMs will be the same in cell types other than fibroblasts. However, we also show here that many of the ESM metabolites change over time following the induction of IrrDSBsen in line NHOF-1 which does not express p16^INK4A^, suggesting that the ESM metabolites might be good biomarkers of both p16^INK4A^-independent senescence as well of p16^INK4A^-dependent senescence.

Several IrrDSBsen metabolites overlap with independent serum metabolite markers of human chronological ageing. However, apart from the PEsen metabolites reported previously all but a few are also elevated in quiescent cells including glutamate, and aspartate which are independent indicators of chronological age in humans^23^. Amongst the new metabolites identified 3-hydroxyisobutyrate and isovalerate are downstream breakdown products of the BCAA pathway, which has been implicated in ageing^42,43^ and age-related disease^44^. In *c*. *elegans* increased levels of BCAAs are associated with longevity^42^ and so the upregulation of 3-hydroxyisobutyrate and isovalerate might be indicative of increased BCAA catabolism in senescent cells, which in turn could contribute to age-related diseases reviewed in^45^. BCAA catabolism is highest in the brain, kidney and cardiac muscle which are the organs of highest importance. An excess of BCAAs can lead to neuronal dysfunction in Maple syrup urine disease and certain cardiomyopathies. Furthermore there is evidence that BCAA catabolism is essential for normal cardiac physiology and viability. Therefore, the increase in BCAA catabolites in the senescent fibroblast ESM might be to provide energy to senescent cells with dysfunctional mitochondria similar to the increase of glycolytic and pentose phosphate pathway metabolites reported previously^22^. Alpha lipoate is an antioxidant and is depleted from the medium of IrrDSBsen cells, and aged rats supplemented with alpha lipoic acid in their diet showed a reduced level of lipid peroxides. 2-aminoadipate is an intermediate in the breakdown or degradation of lysine and saccharopine. Aging is known to lead to the oxidation of lysyl residues to 2-aminoadipic acid in human skin collagen and potentially other tissues^46^. Proteolytic breakdown of these tissues by matrix metalloproteinases in the SASP^7^ could lead to the release of free 2-aminoadipic acid and possibly trans-4-hydroxyproline which is also elevated in the ESM of IrrDSBsen cells. Finally, caprylate (8.0) or octanoic acid has no obvious link to ageing but may be related to beta hydroxybutyrate metabolism and mitochondrial function.

Extracellular citrate (EC) is one of the most consistent and specific markers of PEsen and IrrDSBsen (see above). Therefore, we examined the kinetics of its induction by targeted metabolite analysis in two cell lines. We showed that whilst EC was slightly elevated when IrrDSBs are established at day 5 after irradiation, it generally began to accumulate at day 10 and remained high at day 20, supporting previous data that it is a marker of the established senescent phenotype rather than cell cycle arrest or repairable DSBs^22^. We also established that p16^INK4A^ is not required for the induction of EC as it was absent from the NHOF-1 cells used in the studies reported here. The absence of p16^INK4A^ might affect the kinetics of its induction somewhat as both NHOF-5 and IMR90 accumulated EC by day 10, whereas NHOF-1 accumulated EC a bit more slowly in both the targeted and untargeted analyses. However, targeted knock down of p16^INK4A^ in isogenic lines is required to test this hypothesis.

Blood citrate levels have been shown to increase with chronological age in humans^23,24^ and in age-related diseases such as non-alcoholic fatty acid disease and type 2 diabetes where senescent cells are known to be involved in the development of the disease^25,26,28^. However, both of these age-related diseases are associated with metabolic syndrome and other tricarboxyllic acid cycle metabolites such as succinate, malate, alpha ketoglutarate and fumarate also increase in the blood in addition to citrate^26^. In contrast, only citrate increases in the ESM^22^ and citrate is much more strongly associated with human chronological age than any other tricarboxyllic acid cycle metabolite^23^. Therefore, at present it is unclear whether the elevated levels of blood citrate in human age-related diseases are due to the increased numbers of senescent cells. Finally, citrate is required for the optimum growth of pancreatic xenografts and a variant of the mitochondrial plasma membrane transporter SLC25A1/CiC, termed pmCiC^47^ appears to be essential and is upregulated on the surface of a wide variety of human cancers^47^. The authors suggested that senescent human fibroblasts could be one of the sources of EC in human cancers^47^ and as many malignancies^48–50^ and premalignancies^9,50^ are associated with senescent cancer-associated fibroblasts this could well be the case. However, this hypothesis requires experimental verification.

Although there is a considerable overlap between the PEsen and IrrDSBsen ESMs and human serum ageing signatures, many metabolites that associate with ageing do not appear in the ESM. Other biomarkers of ageing may have nothing to do with senescent cells, may be produced by cell types other than fibroblasts or by other mechanisms of senescence that are independent of IrrDSBs. In addition, our *in vitro* work was conducted in high levels of glucose and atmospheric oxygen with only 10% of the serum proteins present *in vivo*, so it may be that there is more overlap between ageing and cellular senescence *in vivo* than we have detected. Why do some ESM metabolites not show up in ageing signatures? One answer may be that unbiased screens often miss metabolites. For example, 3- hydroxyisobutyrate was detected in only two out of four of the screens we conducted, and this metabolite did not appear in the human chronological ageing screen conducted by Menni *et al*. using the same service provider and techniques^23^.

## Conclusions

Firstly, the IrrDSBsen ESM is a dynamic process but unlike the recently published transcriptomic analysis of IrrDSBsen^31^ most ESM metabolites are induced by day 5 or 10 and remain high for the 20 day study period. Whilst a few metabolites did become transiently altered and then return to normal, with the exception of asparagine and glutamate, this did not explain their omission from the PEsen ESM published previously. Secondly, human serum metabolites that are associated with ageing are also present in the IrrDSBsen and PEsen fibroblast ESMs but most may be related to the cell cycle arrest phase of the senescent phenotype rather than the later stages of the senescent phenotype. Finally, EC, one of the most consistently observed and specific ESM metabolites, making it a good marker of senescent cells, both p16-positive and -negative.

## Experimental Procedures

### Cell Culture

The NHOF-1 and NHOF-5 oral fibroblast lines’ characterisation and the culture methods have been described previously^9,22^. IMR90 lung embryo fibroblasts were obtained from the American Type Culture Collection. Briefly, all fibroblasts were grown in Dulbecco’s Modified Eagles Medium containing 10% vol/vol foetal bovine serum (FBS) in an atmosphere of 10% CO_2_/90% air and subcultured once weekly at a density of 1 × 10^5^ cells per 9 cm plate to prohibit confluence. Mean population doublings (MPDs) were calculated as described previously^38^. Quiescent control cultures were allowed to remain confluent or in 0.1% vol/vol FBS for 4 days before analysis and the medium was changed every day. The PEsen and growing cultures were also medium changed every day.

### Senescence Induction by Gamma Rays

The cells were defined as PEsen by numerous markers^9,22^ and by the extension of replicative lifespan following the retroviral transduction of the catalytic component of telomerase (James and Parkinson – unpublished data).

Fibroblasts were irradiated in suspension with γ rays from a Cs source at a dose rate of 1.4 Gy/min with either 0.5 Gy to induce repairable DNA double strand breaks or 20 Gy to induce irreparable DNA double strand breaks, as described, and the cells were left between 0 and 20 days in culture before analysis.

### Collection of conditioned medium

To collect the conditioned medium, cells were plated in T175 flasks or 9 cm dishes in such a way as to ensure a similar cell density to medium volume ratio at the day of collection. The medium was removed and the cells washed once with fresh medium before adding the appropriate medium. After equilibration in 10% CO_2_/90% air, the flasks or dishes were sealed with Parafilm to prevent evaporation and maintain a constant pH, whilst maintaining a high cell to medium ratio. Cells were counted at the end of the incubation period to obtain an average cell count throughout the collection period. Medium was harvested for analysis after 24 hours.

### Metabolite analysis

Conditioned medium was collected after 24 hours from the cells, centrifuged at 800 × g for 2 minutes, the supernatant removed and centrifuged again at 13,000 rpm for 2 minutes and the final supernatant snap frozen on an ethanol-dry ice bath for 15 minutes before storage at −80 °C. Unconditioned medium was also prepared identically.

### Unbiased metabolomics: Metabolomic analysis, normalisation and data presentation as scaled intensity

The unbiased metabolomics analysis was carried out by Metabolon Inc. The details of the metabolomics analysis have been published previously, including sample preparation, instrumentation, conditions for mass spectrometry (liquid chromatography/tandem mass spectrometry in positive and negative ion modes, and gas chromatography/mass spectrometry), peak data reduction, and assignment of peaks to known chemical entities by comparison to metabolite library entries of purified standards, has been previously described^22^. Briefly, for analysis, the median signal intensity of a given biochemical was determined across all sample groups. This median was subsequently used to scale individual samples to a median of 1 for the group. A minimum value was assigned when a biochemical was not detected in an individual sample (this was rare). This data is graphically presented as scaled intensity and is thus a measure of the relative level of each metabolite. For further details see refs^22,31^).

### Targeted measurement of extracellular citrate by gas chromatography/mass spectroscopy (GCMS)

Deuterated citrate (d4Citrate) was added to each sample to a final concentration of 0.1 mM as an internal standard. Metabolites were then extracted using cold methanol before being dried under vacuum desiccation. The samples were re-suspended in anhydrous pyridine containing the derivitisation agents methoxyamine hydrochloride followed by N-Methyl-N-trimethylsilyltrifluoroacetamide with 1% 2,2,2-Trifluoro-N-methyl-N-(trimethylsilyl)-acetamide, Chlorotrimethylsilane (MSTFA + 1%TMCS). GCMS was performed in pulsed splitless mode on a Hewlett Packard HP6890 series GC system with Agilent 6890 series injector and a 30 m long 250 µm diameter capillary column (Agilent, model number 19091s-433HP5MS) using a flow rate of 1 mL/minute, and a Hewlett Packard 5973 Mass selective detector. The acquisition was conducted in selective ion monitoring mode, the ion masses detected for citrate were: 273, 347, 375 and 465 and the corresponding heavy ions were 276, 350, 378 and 469. The dwell time for all of these ions was 50 ms.

### Cell characterisation

SA-βGal activity was performed using the senescence detection kit from Biovision^9^. The immunofluorescent quantification of ki67 and large 53BP1 foci has been described in previous publications along with the relevant controls^22,39^.

Western blotting was carried out as described previously^51^ using the following antibodies: p16^INK4A^ (Anti-CDKN2A/p16INK4a EP4353Y(3) ab81278 from Abcam, UK), MCM7 (Anti-MCM7 EP1974Y ab52489 from Abcam, UK) and β-actin. All antibodies were diluted in blocking buffer (5% ilk in TBS-T) at 1:2000 (MCM7 and p16^INK4A^) and 1:20,000 (β-actin) and incubated with the membrane overnight at 4 °C, under gentle agitation. HeLa cell extracts were used as positive controls. Cell lines lacking p16^INK4A^ were used as negative controls in other experiments (Karen Ng Lee Peng – unpublished data).

### Statistical analysis

A two-sample unpaired *T*-test was used to identify biochemicals that differed significantly between experimental groups in the unbiased metabolomic screens. For the characterisation of the IrrDSBsen and PEsen cultures and the targeted analysis of citrate, one way analysis of variance (ANOVA) and Tukey’s *post hoc* test were used.

### Data availability

We have submitted our ‘Metabolon Inc.’ files including the explanation of the data, the statistical analysis using the Welch’s Test, the raw data, the crude heat maps and the pathway-sorted box plots to the Dryad Data Repository Provisional DOI: doi:10.5061/dryad.45747rs Data files: Metabolomic changes during radiation-induced senescence in oral fibroblasts.

## Electronic supplementary material

Supplementary Information
