# Supplementary material for: The induction of the fibroblast extracellular senescence metabolome is a dynamic process
Source: Sci Rep. 2018 Aug 14;8:12148. doi: 10.1038/s41598-018-29809-5 (PMC6092376; doi:10.1038/s41598-018-29809-5)
Supplement: Supplementary file 1 — Supplementary Information [file 41598_2018_29809_MOESM1_ESM.pdf]

The induction of the fibroblast extracellular senescence metabolome is a dynamic process.

Emma N. L. James, Mark H. Bennett[1] and E. Kenneth Parkinson[2]

Centre for Clinical & Diagnostic Oral Sciences, Institute of Dentistry, Barts and the London School of Medicine and Dentistry, Queen Mary University of London, Turner Street, London E1 2AD, UK

[1]Department of Life Science, South Kensington Campus, Imperial College London, London SW7 2AZ, UK

[2]Correspondence to: Eric Kenneth Parkinson, Centre for Immunobiology and Regenerative Medicine, Blizard Institute, 4 Newark Street, London E1 2AT UK, Phone: 44-(0)207-882-7185 Fax: 44(0)207-882-7137, Email: e.k.parkinson@qmul.ac.uk

Running Title: The dynamics of the extracellular senescence metabolome

Key words: Extracellular, senescence, metabolome, DNA, strand breaks, dynamics, chronological ageing.

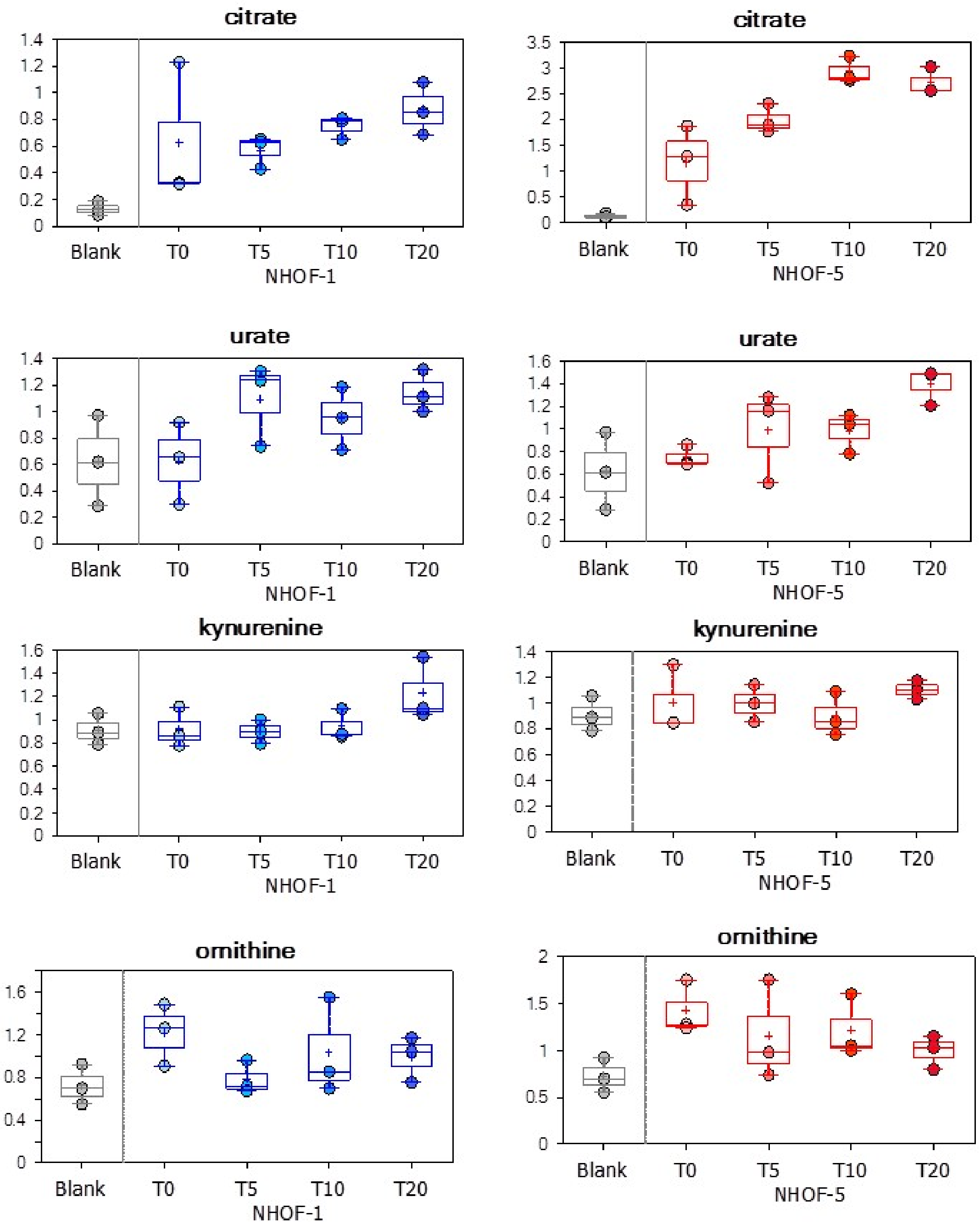
citrate
1.4
1.2
1
0.8
0.6
0.4
0.2
0
Blank
T0
T5
T10
T20
NHOF-1
citrate
3.5
3
2.5
2
1.5
1
0.5
0
Blank
T0
T5
T10
T20
NHOF-5
urate
1.4
1.2
1
0.8
0.6
0.4
0.2
0
Blank
T0
T5
T10
T20
NHOF-1
urate
1.6
1.4
1.2
1
0.8
0.6
0.4
0.2
0
Blank
T0
T5
T10
T20
NHOF-5
kynurenine
1.6
1.4
1.2
1
0.8
0.6
0.4
0.2
0
Blank
T0
T5
T10
T20
NHOF-1
kynurenine
1.4
1.2
1
0.8
0.6
0.4
0.2
0
Blank
T0
T5
T10
T20
NHOF-5
ornithine
1.6
1.2
0.8
0.4
0
Blank
T0
T5
T10
T20
NHOF-1
ornithine
2
1.5
1
0.5
0
Blank
T0
T5
T10
T20
NHOF-5

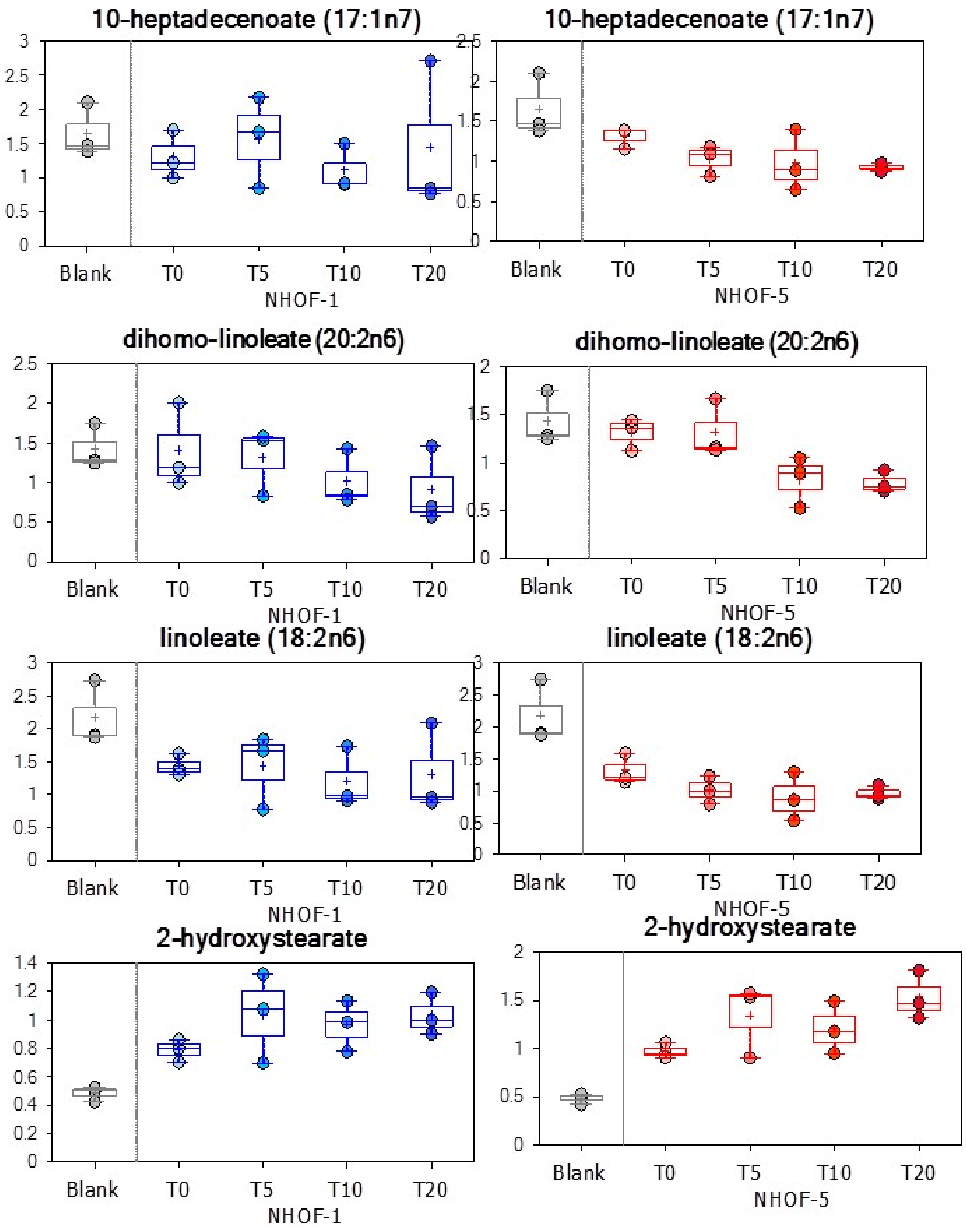
10-heptadecenoate (17:1n7)
Blank
T0
T5
T10
T20
NHOF-1
10-heptadecenoate (17:1n7)
NHOF-5
dihomo-linoleate (20:2n6)
dihomo-linoleate (20:2n6)
linoleate (18:2n6)
linoleate (18:2n6)
2-hydroxystearate
2-hydroxystearate

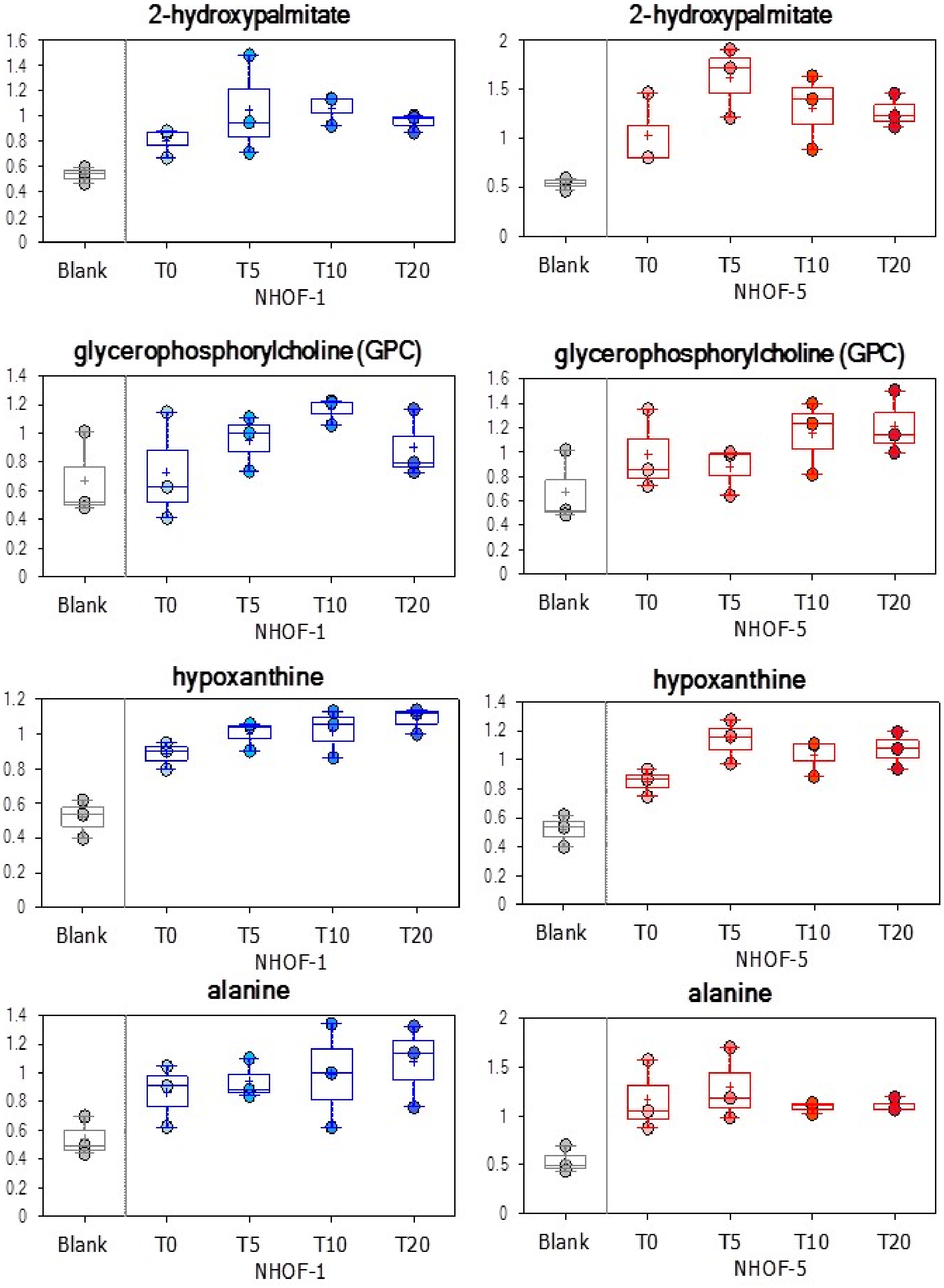
2-hydroxypalmitate
0
0.2
0.4
0.6
0.8
1
1.2
1.4
1.6
Blank
T0
T5
T10
T20
NHOF-1
2-hydroxypalmitate
0
0.5
1
1.5
2
Blank
T0
T5
T10
T20
NHOF-5
glycerophosphorylcholine (GPC)
0
0.2
0.4
0.6
0.8
1
1.2
1.4
Blank
T0
T5
T10
T20
NHOF-1
glycerophosphorylcholine (GPC)
0
0.2
0.4
0.6
0.8
1
1.2
1.4
1.6
Blank
T0
T5
T10
T20
NHOF-5
hypoxanthine
0
0.2
0.4
0.6
0.8
1
1.2
Blank
T0
T5
T10
T20
NHOF-1
hypoxanthine
0
0.2
0.4
0.6
0.8
1
1.2
1.4
Blank
T0
T5
T10
T20
NHOF-5
alanine
0
0.2
0.4
0.6
0.8
1
1.2
1.4
Blank
T0
T5
T10
T20
NHOF-1
alanine
0
0.5
1
1.5
2
Blank
T0
T5
T10
T20
NHOF-5

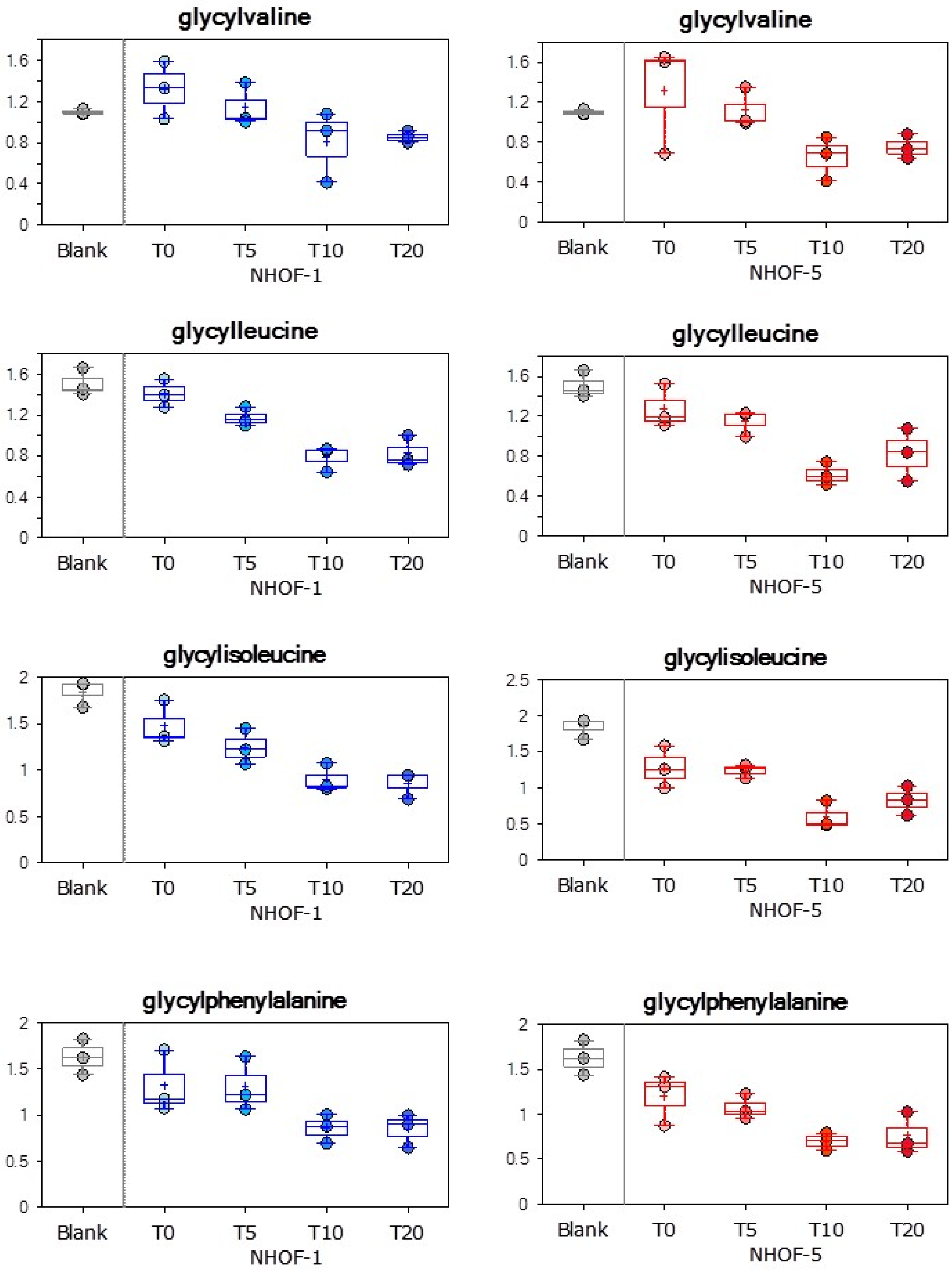
glycylvaline
1.6
1.2
0.8
0.4
0
Blank
T0
T5
T10
T20
NHOF-1
glycylvaline
1.6
1.2
0.8
0.4
0
Blank
T0
T5
T10
T20
NHOF-5
glycylleucine
1.6
1.2
0.8
0.4
0
Blank
T0
T5
T10
T20
NHOF-1
glycylleucine
1.6
1.2
0.8
0.4
0
Blank
T0
T5
T10
T20
NHOF-5
glycylisoleucine
2
1.5
1
0.5
0
Blank
T0
T5
T10
T20
NHOF-1
glycylisoleucine
2.5
2
1.5
1
0.5
0
Blank
T0
T5
T10
T20
NHOF-5
glycylphenylalanine
2
1.5
1
0.5
0
Blank
T0
T5
T10
T20
NHOF-1
glycylphenylalanine
2
1.5
1
0.5
0
Blank
T0
T5
T10
T20
NHOF-5

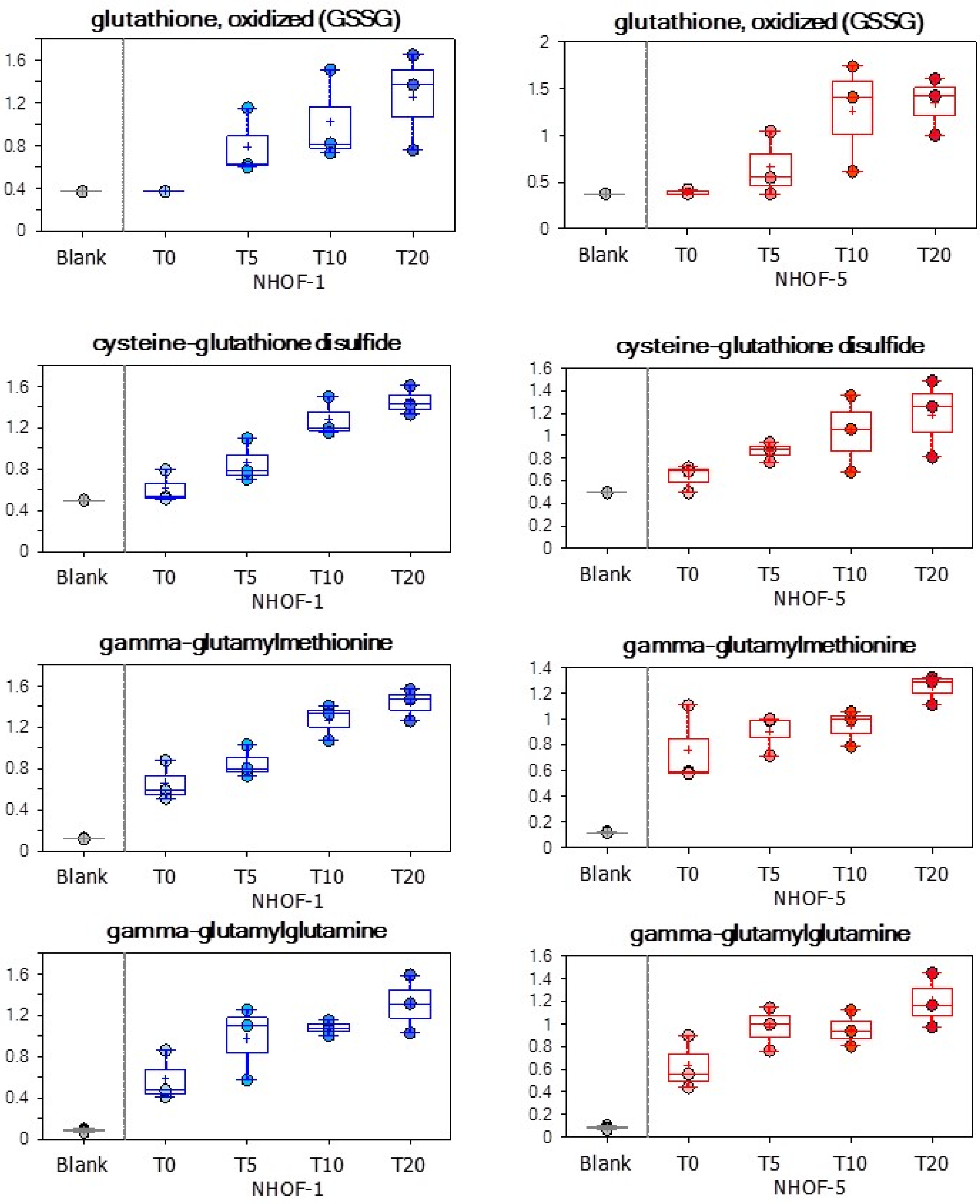

glutathione, oxidized (GSSG)
0
0.4
0.8
1.2
1.6
Blank
T0
T5
T10
T20
NHOF-1
glutathione, oxidized (GSSG)
0
0.5
1
1.5
2
Blank
T0
T5
T10
T20
NHOF-5
cysteine-glutathione disulfide
0
0.4
0.8
1.2
1.6
Blank
T0
T5
T10
T20
NHOF-1
cysteine-glutathione disulfide
0
0.2
0.4
0.6
0.8
1
1.2
1.4
1.6
Blank
T0
T5
T10
T20
NHOF-5
gamma-glutamylmethionine
0
0.4
0.8
1.2
1.6
Blank
T0
T5
T10
T20
NHOF-1
gamma-glutamylmethionine
0
0.2
0.4
0.6
0.8
1
1.2
1.4
Blank
T0
T5
T10
T20
NHOF-5
gamma-glutamylglutamine
0
0.4
0.8
1.2
1.6
Blank
T0
T5
T10
T20
NHOF-1
gamma-glutamylglutamine
0
0.2
0.4
0.6
0.8
1
1.2
1.4
1.6
Blank
T0
T5
T10
T20
NHOF-5

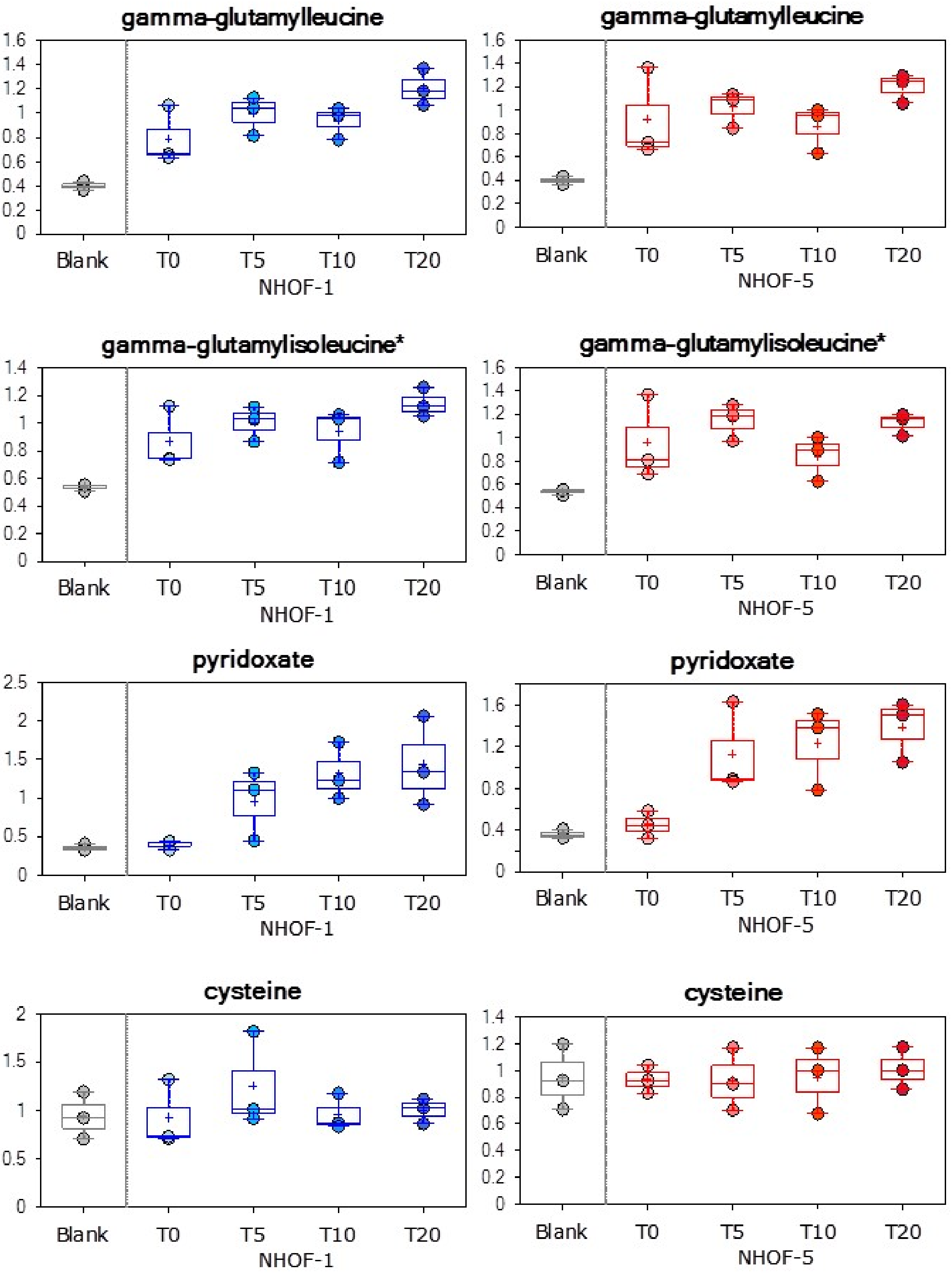
gamma-glutamylleucine
1.6
1.4
1.2
1
0.8
0.6
0.4
0.2
0
Blank
T0
T5
T10
T20
NHOF-1
gamma-glutamylleucine
1.6
1.4
1.2
1
0.8
0.6
0.4
0.2
0
Blank
T0
T5
T10
T20
NHOF-5
gamma-glutamylisoleucine*
1.4
1.2
1
0.8
0.6
0.4
0.2
0
Blank
T0
T5
T10
T20
NHOF-1
gamma-glutamylisoleucine*
1.6
1.4
1.2
1
0.8
0.6
0.4
0.2
0
Blank
T0
T5
T10
T20
NHOF-5
pyridoxate
2.5
2
1.5
1
0.5
0
Blank
T0
T5
T10
T20
NHOF-1
pyridoxate
1.6
1.2
0.8
0.4
0
Blank
T0
T5
T10
T20
NHOF-5
cysteine
2
1.5
1
0.5
0
Blank
T0
T5
T10
T20
NHOF-1
cysteine
1.4
1.2
1
0.8
0.6
0.4
0.2
0
Blank
T0
T5
T10
T20
NHOF-5

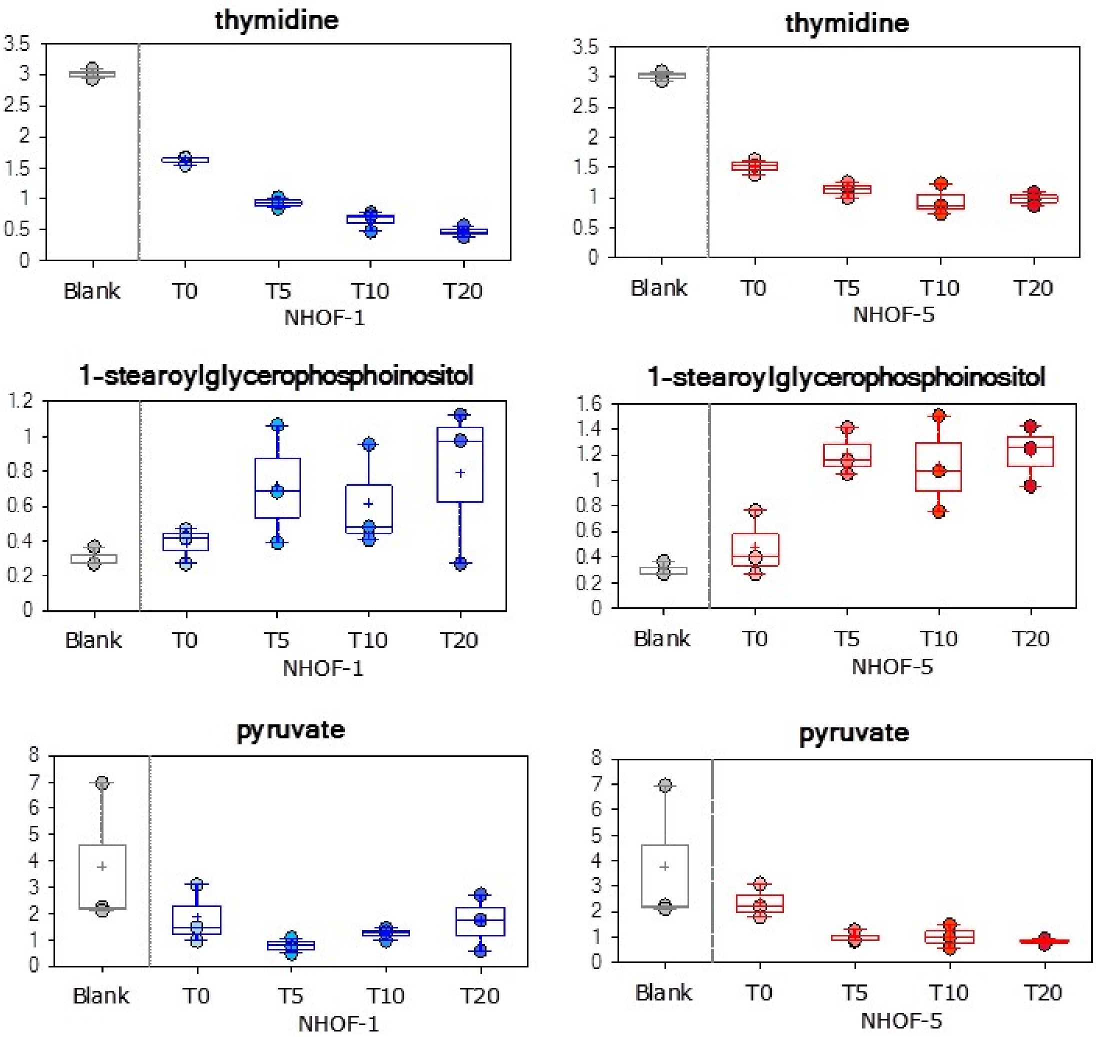
thymidine
Blank
T0
T5
T10
T20
NHOF-1
thymidine
NHOF-5
1-stearoylglycerophosphoinositol
NHOF-1
1-stearoylglycerophosphoinositol
NHOF-5
pyruvate
NHOF-1
pyruvate
NHOF-5

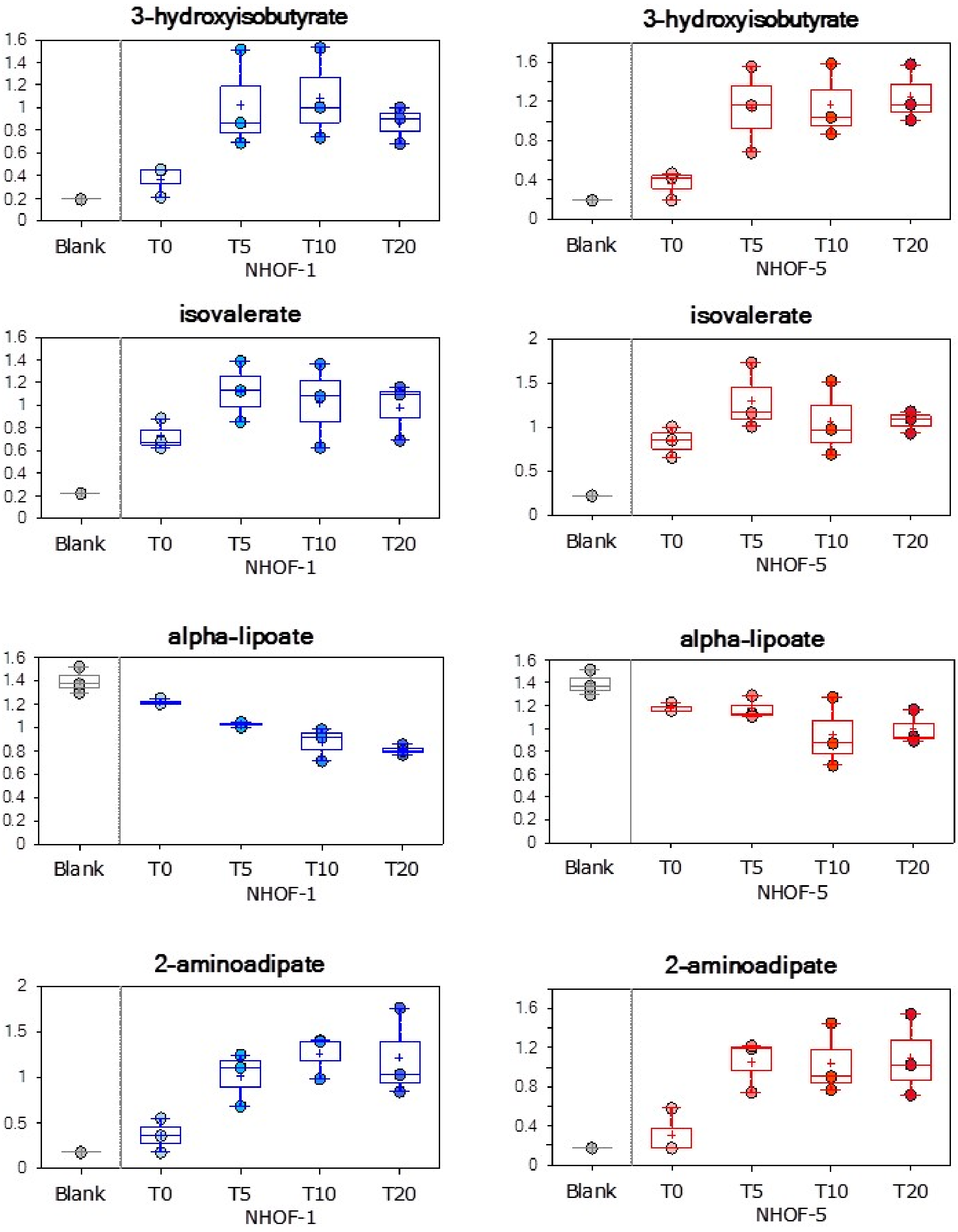
3-hydroxyisobutyrate
Blank
T0
T5
T10
T20
NHOF-1
NHOF-5
isovalerate
alpha-lipoate
2-aminoadipate

Supplementary Figure S1 The kinetics of the induction of the IrrDSBsen

The first 7 pages show the time course of each detectable ESM PEsen metabolite after the induction of IrrDSBsen over a 20 day period in two oral fibroblast lines NHOF-1 and NHOF-5. The last four panels show the same for four IrrDSBsen metabolites not previously identified in the PEsen ESM. The symbols for the box and whisker plots are as follows; + = mean value, the horizontal lines within the boxes = median value, O = extreme points, the limits of the boxes = the upper and lower quartiles and the error bars indicate the maxima and minima of distribution.

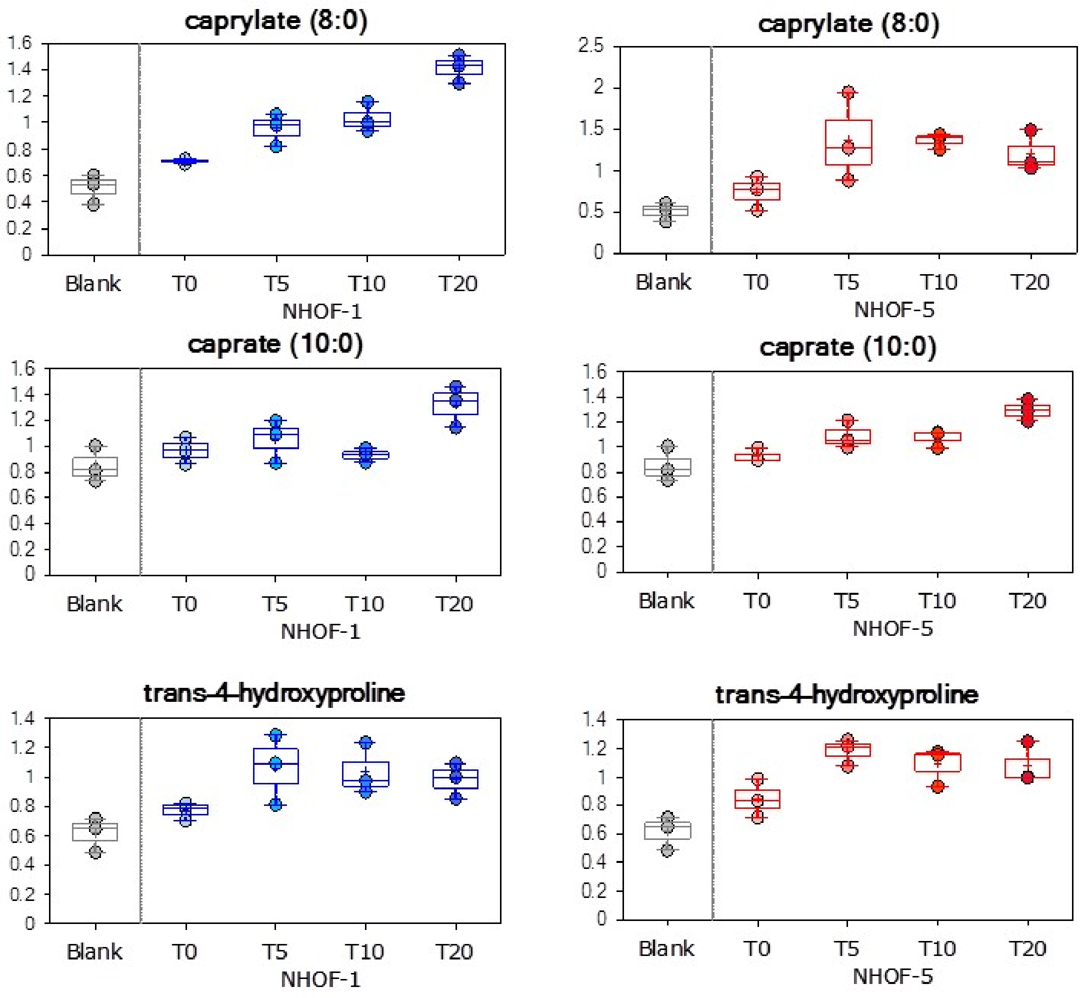

Supplementary Figure S2 The kinetics of the induction of the IrrDSBsen

The figure shows the time course of each detectable metabolite previously reported to be associated with chronological ageing (see reference 21) not previously detected in the PEsen ESM after the induction of IrrDSBsen over a 20 day period in two oral fibroblast lines NHOF-1 and NHOF-5. The symbols for the box and whisker plots are the same as for supplementary Figure S1.

A

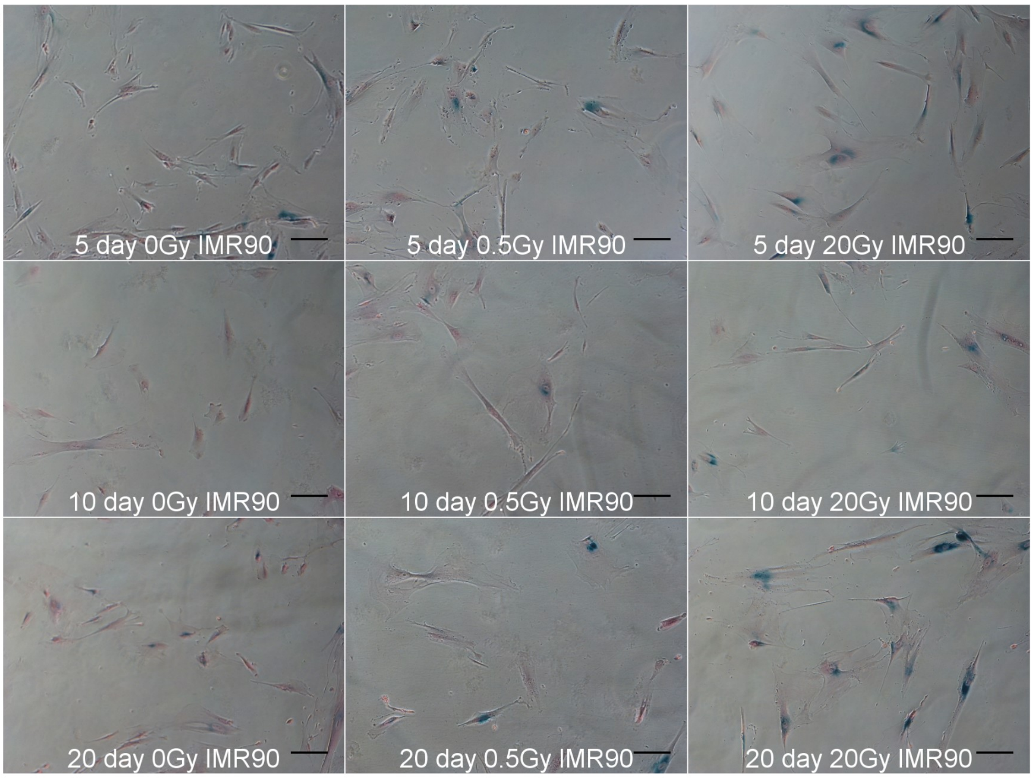

B

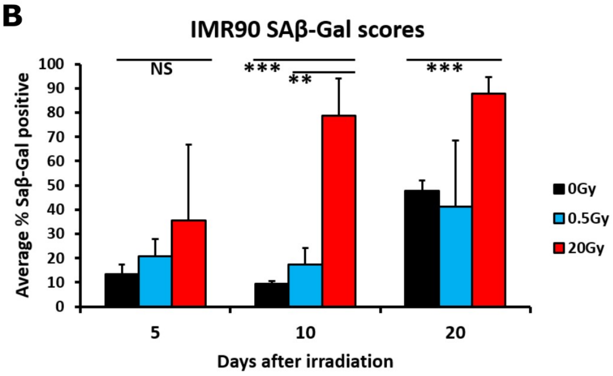

Supplementary Figure S3 SAβ-Gal staining of IrrDSBsen IMR90 cells and controls. **A** Representative SAβ-gal staining of 0Gy 0.5Gy and 20Gy treated IMR90 at 5, 10 and 20 days after treatment. Cells counterstained with nuclear fast red. Scale bar represents 50µm. **B** Average % SAβ-Gal scores 0Gy 0.5Gy and 20Gy treated IMR90 at 5, 10 and 20 days after treatment. Error bars represent standard deviation from the mean. N=3 NS = non significant, **p<0.01***p<0.001 with 1 way ANOVA and Tukey's post hoc analysis.

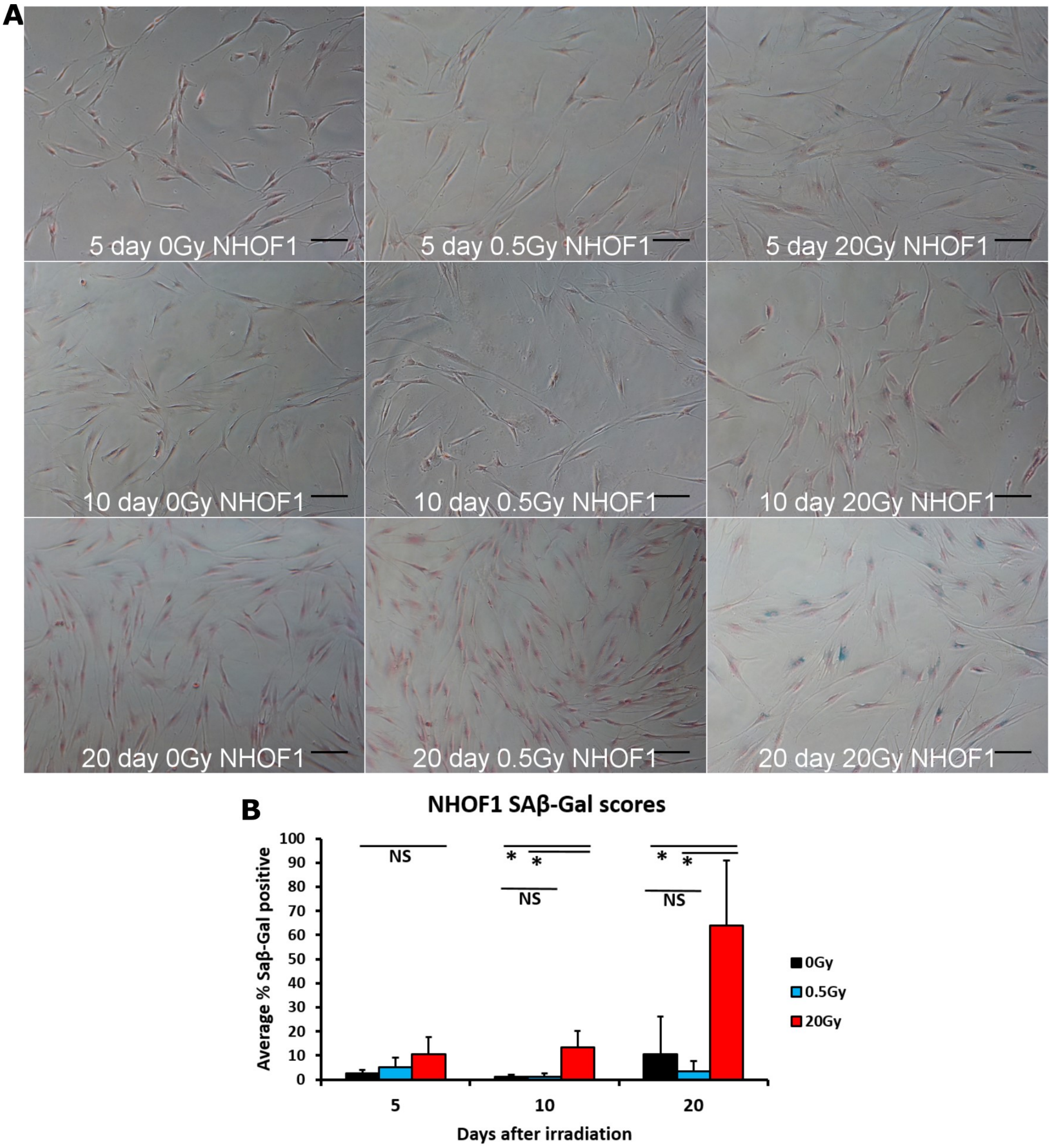

Supplementary Figure S4 SAβ-Gal staining of IrrDSBsen NHOF-1 cells and controls.

A Representative SAβ-gal staining of 0Gy 0.5Gy and 20Gy treated NHOF-1at 5, 10 and 20 days after treatment. Cells counter stained with nuclear fast red. Scale bar represents 50μm. **B** Average % SAβ-Gal scores 0Gy 0.5Gy and 20Gy treated NHOF-1 at 5, 10 and 20 days after treatment. Error bars represent standard deviation from the mean. N=3 NS = non significant, *p<0.05 with 1 way ANOVA and Tukey's post hoc analysis.

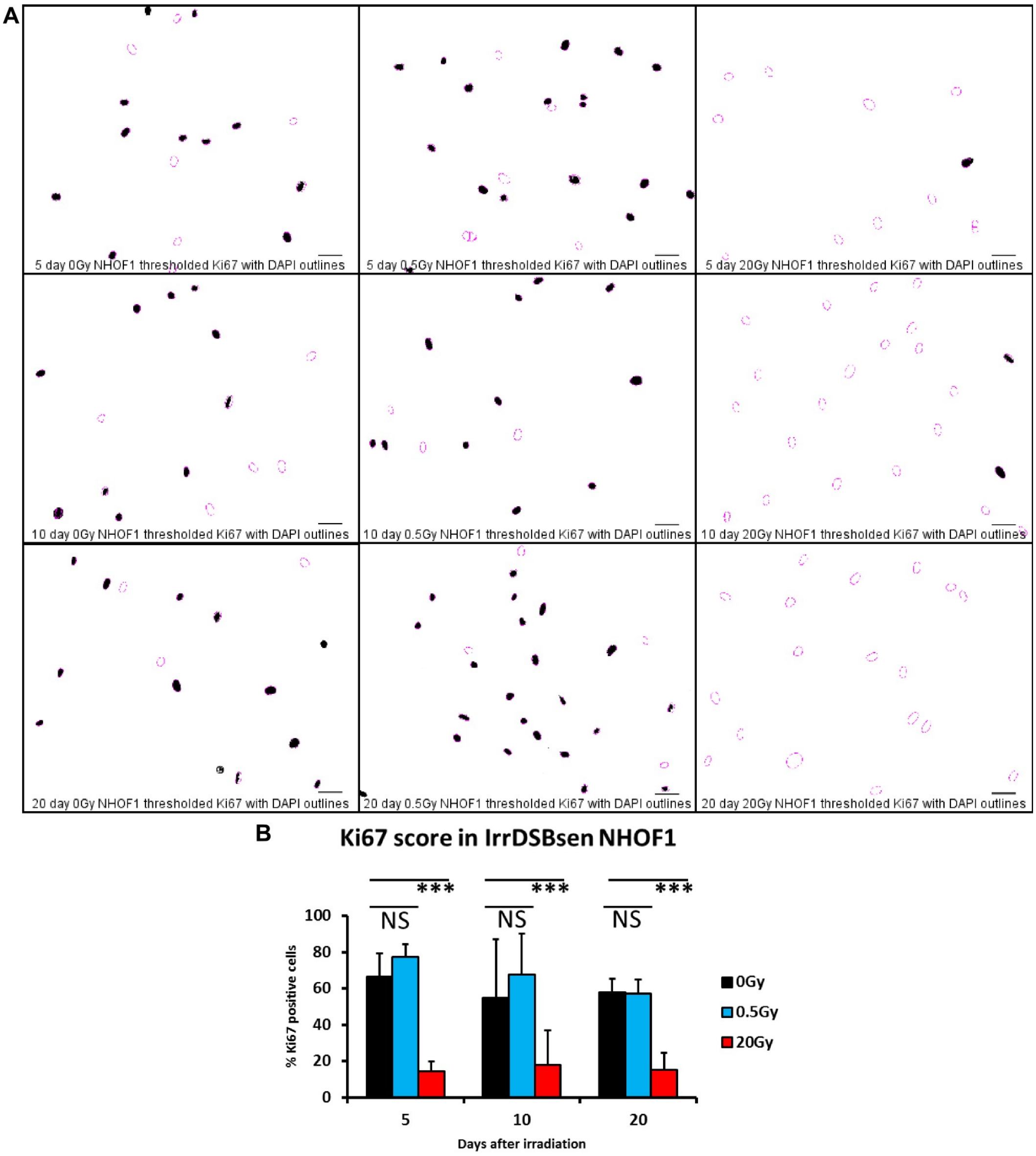

Supplementary Figure S5 Ki67 staining of IrrDSBsen NHOF-1 and controls.

A Thresholded image of Ki67 staining of NHOF-1 cells 5, 10 and 20 days after exposure to 0Gy, 0.5Gy and 20Gy. Staining and image acquisition protocols are described in supplementary method 1. Nuclear regions of interest (identified using DAPI) are shown as magenta outlines. Scale bar represents 50µm. **B** Mean Ki67 scores for each group across three repeats. Counts were obtained using ImageJ, by the method described in supplementary method 2. Error bars represent standard deviation from the mean, n=3

NS= not significant ***p<0.001 with 2 way ANOVA using Tukey's post hoc analysis.

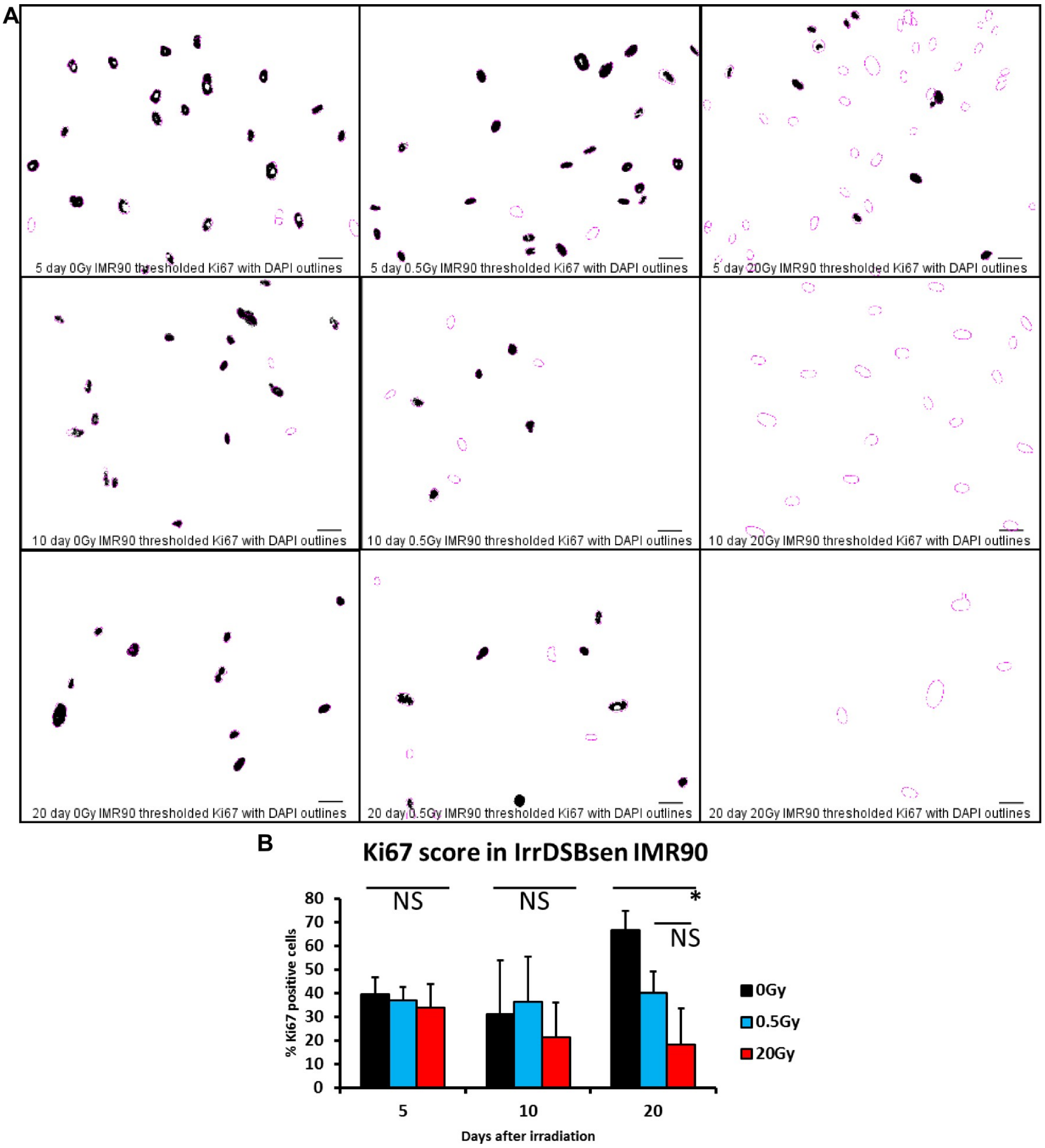

Supplementary Figure S6 Ki67 staining of IrrDSBsen IMR90 and controls.

A Thresholded Ki67 staining in IMR90 cells 5, 10 and 20 days after a 0, 0.5 and 20Gy dose ionising radiation. Staining and image acquisition protocols are described in supplementary method 1. Nuclear regions of interest (identified using DAPI) are shown as magenta outlines. Scale bar represents 50µm. **B** Mean Ki67 scores for each group across three repeats. Counts were obtained using ImageJ, by the method described in

supplementary method. Error bars represent standard deviation from the mean. NS = not significant *$p<0.05$, 2 way ANOVA with Tukey's post hoc analysis.

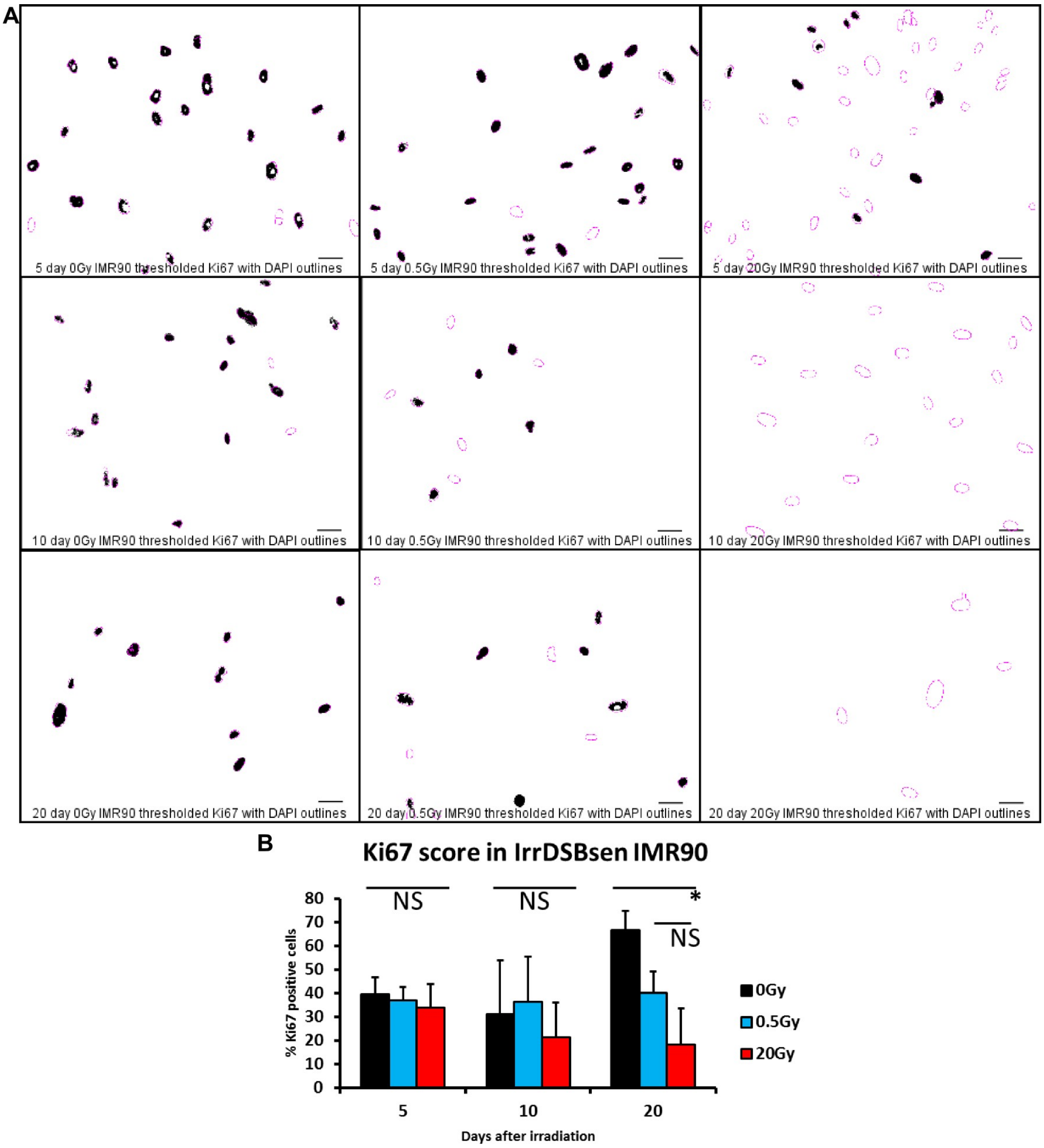

Supplementary Figure S7 Ki67 staining of IrrDSBsen IMR90 and controls.

A Thresholded Ki67 staining in IMR90 cells 5, 10 and 20 days after a 0, 0.5 and 20Gy dose ionising radiation. Staining and image acquisition protocols are described in supplementary method 1. Nuclear regions of interest (identified using DAPI) are shown as magenta outlines. Scale bar represents 50µm. **B** Mean Ki67 scores for each group across three repeats. Counts were obtained using ImageJ, by the method described in

supplementary method 2. Error bars represent standard deviation from the mean. NS = not significant *p<0.05, 2 way ANOVA with Tukey's post hoc analysis.

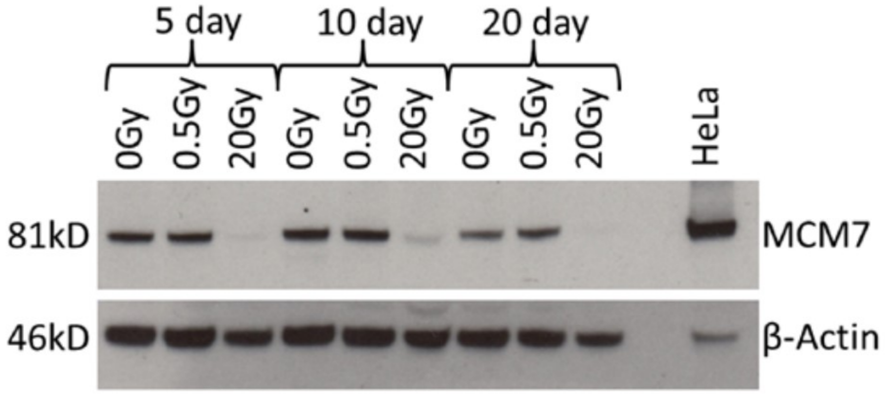

Supplementary Figure S8 MCM7 protein levels in IrrDSBsen IMR90 and controls.

Representative western blot showing MCM7 protein levels in IMR90 cells treated with 0Gy, 0.5Gy and 20Gy on days 5, 10 and 20 after treatment. Blots were performed separately on lysates from three independent experiments. HeLa is a positive control. White space between rows of bands depicts where the membrane has been stripped and re-probed. The exposure times may be different for each section of membrane. Uncropped images of films used to make this figure can be seen in supplementary figures S18A and S18B.

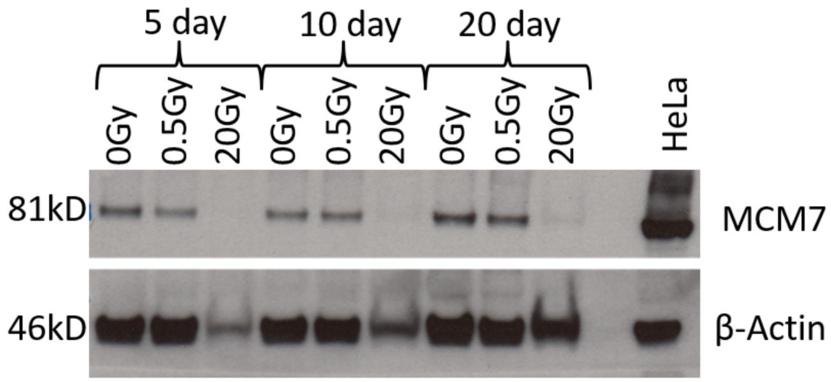

Supplementary Figure S9 MCM7 protein levels in IrrDSBsen NHOF-1 and controls.

Representative western blot showing MCM7 protein levels in NHOF-1 cells treated with 0Gy, 0.5Gy and 20Gy on days 5, 10 and 20 after treatment. Blots were performed separately on lysates from three independent experiments. HeLa is a positive control. White space between rows of bands depicts where the membrane has been stripped and re-probed. The exposure times may be different for each section of membrane. Uncropped images of films used to make this figure can be seen in supplementary figures S18C and S18D.

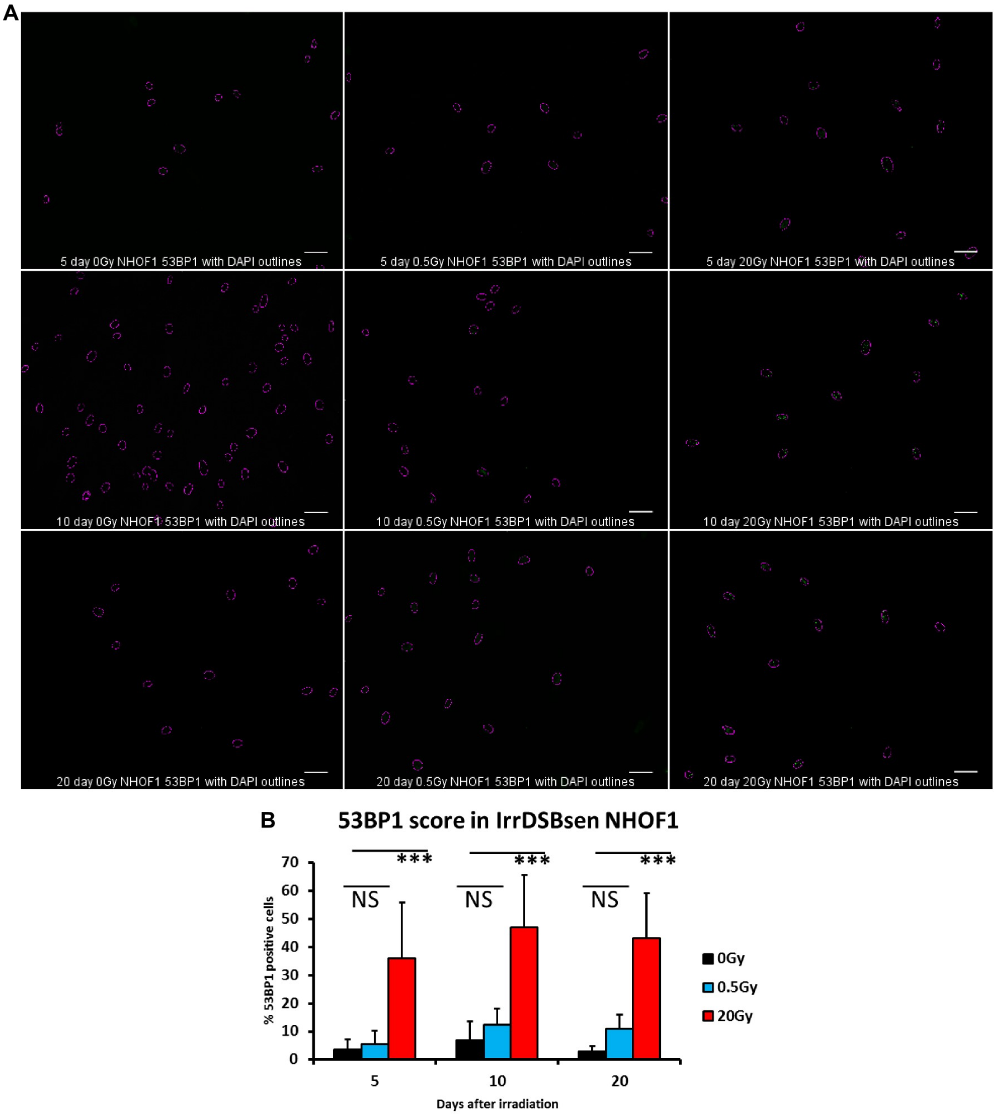

Supplementary Figure S10 53BP1 staining in IrrDSBsen NHOF-1 cells and controls.

A ImageJ nuclear overlays (magenta dotted lines) on 53BP1 foci (green) in NHOF1 after 0, 0.5 or 20Gy ionizing radiation on 5, 10 and 20 day time points. Scale bar represents 50µm. Staining and image acquisition protocols are described in supplementary method 1 **B** shows the mean score for each group across three repeats. Counts were obtained using ImageJ, by the method described in supplementary method 3. Error bars represent standard deviation from the mean. NS = not significant ***p<0.001, 2 way

ANOVA with Tukey's post hoc analysis.

A

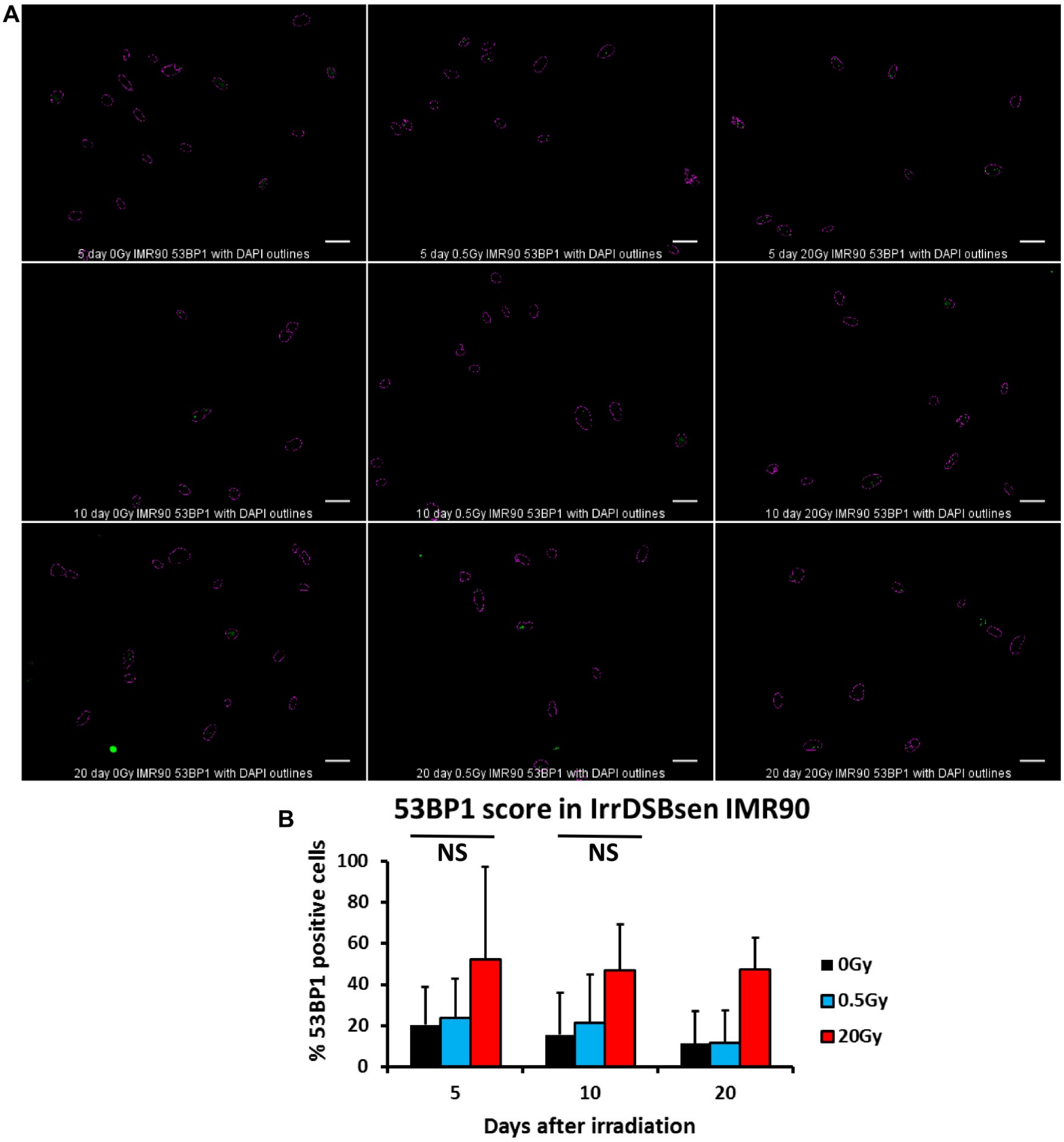

Supplementary Figure S11 53BP1 staining in IrrDSBsen IMR90 and controls.

A ImageJ nuclear overlays (magenta dotted lines) on 53BP1 foci (green) in IMR90 after 0, 0.5 or 20Gy ionizing radiation on 5, 10 and 20 day time points. Scale bar represents 50µm. Staining and image acquisition protocols are described in supplementary method 1 **B** shows the mean score for each group across three repeats. Counts were obtained using ImageJ, by the method described in supplementary method 3. Error bars represent standard deviation from the mean, n=3 in 5 and 10 day groups, n=2 in day 20

group due to damage on the slide. NS = not significant with 1 way ANOVA.

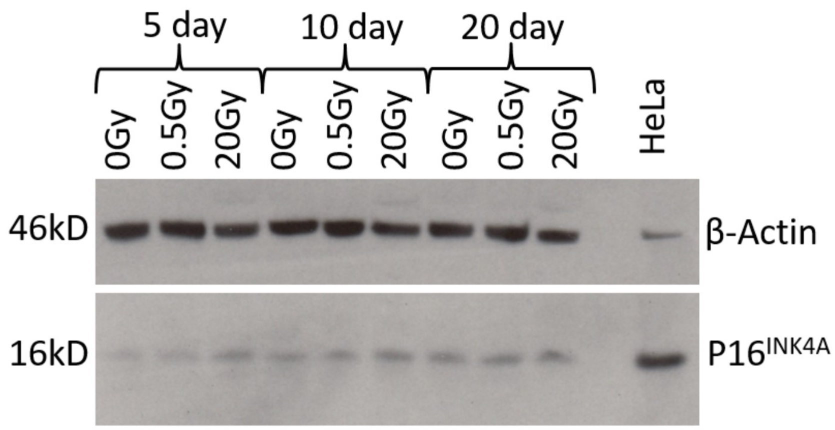

Supplementary Figure S12 p16^{INK4A} protein levels in IrrDSBsen IMR90 and controls.

Representative western blot of IMR90 lysate showing a slight increase in p16^{INK4A} levels over time and with 20Gy gamma irradiation, and the loading control β-actin. Blots were performed separately on lysates from three independent experiments. HeLa is a positive control. White space between rows of bands depicts where the membrane has been cut for incubation with different primary antibodies. The exposure times may be different for each section of membrane. Uncropped images of films used to make this figure can be seen in supplementary figures S18E and S18F.

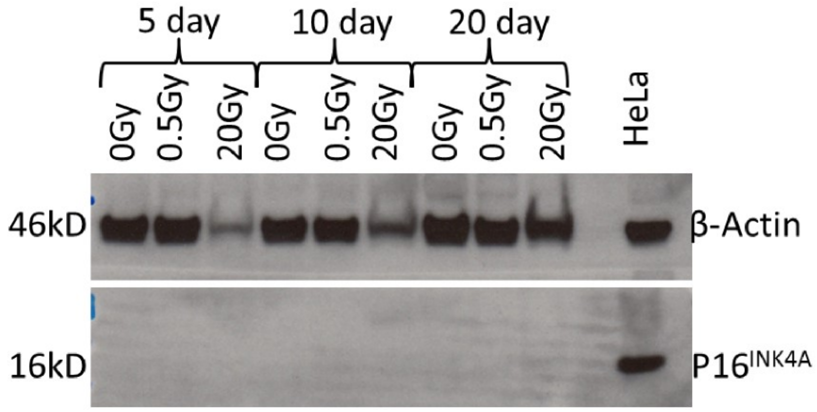

Supplementary Figure S13 p16^{INK4A} protein levels in IrrDSBsen NHOF1 and controls. Representative western blot of p16^{INK4A}protein levels in IrrDSBsen NHOF1 cells. Blots were performed separately on lysates from three independent experiments. HeLa is a positive control. White space between rows of bands depicts where the membrane has been cut for incubation with different primary antibodies. The exposure times may be different for each section of membrane. Uncropped images of films used to make this figure can be seen in supplementary figure S18D.

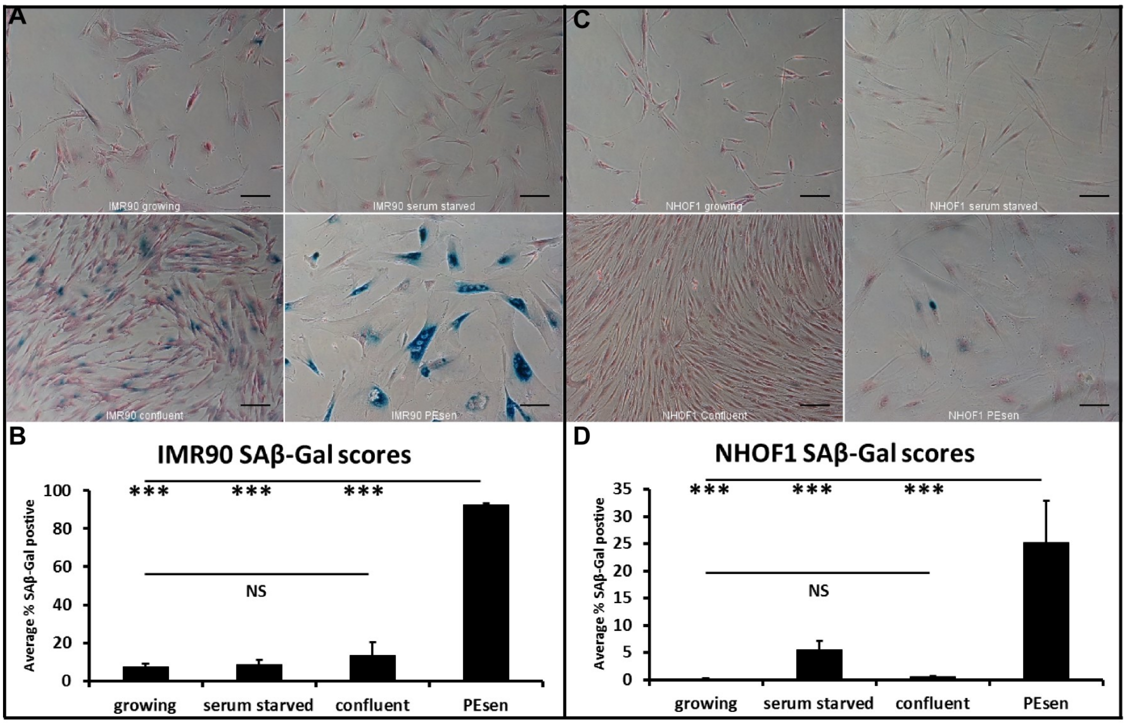

Supplementary Figure S14 SAβ-Gal staining of PEsen and growth arrest controls. Top panels show representative images of growing, serum starved, confluent and PEsen IMR90 (A) and NHOF-1 (C) cells treated with X-gal; blue pigment has accumulated in cells with high beta galactosidase activity. Cells counter stained with nuclear fast red. Scale bars represent 50mm. Bottom panels show average % cells staining positive for SAβ-Gal in IMR90 (B) and NHOF-1 (D). n=3 NS: not significant ***p<0.01 with 1 way ANOVA, Tukey's post hoc analysis.

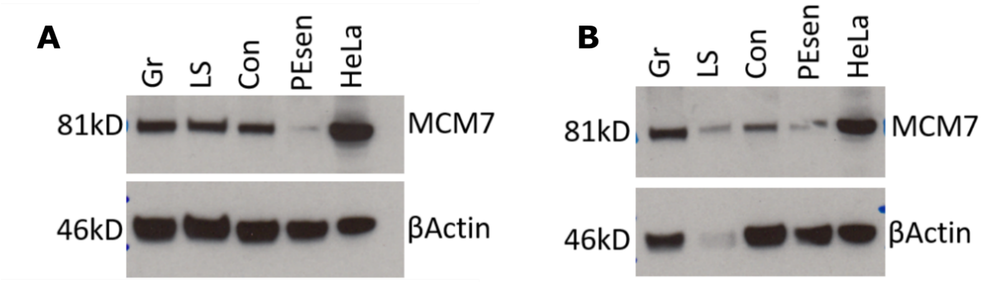

Supplementary Figure S15 MCM7 protein level in PEsen fibroblasts.

Representative western blots of MCM7 protein levels in PEsen, growing (Gr), serum starved (LS) and confluent (Con) controls with β-actin loading control, in IMR90 (**A**) and NHOF-1 (**B**). Blots were performed separately on lysates from three independent experiments. White space between rows of bands depicts where the membrane has been stripped and re-probed. The exposure times may be different for each section of membrane. Uncropped images of films used to make this figure can be seen in supplementary figures S18G and S18H.

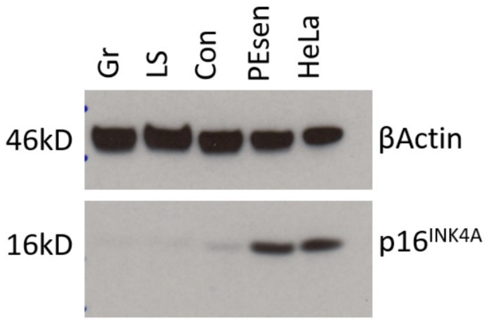

Supplementary Figure S16. p16^{INK4A} protein level in PEsen IMR90 and growth arrest controls.

Representative western blot of p16^{INK4A} protein levels in PEsen IMR90 cells

as well as growing (Gr), serum starved (LS) and confluent (Con) controls, with β-actin loading control. White space between rows of bands depicts where the membrane has been cut for incubation with different primary antibodies. The exposure times may be different for each section of membrane. Uncropped images of films used to make this figure can be seen in supplementary figures S18H and S18I.

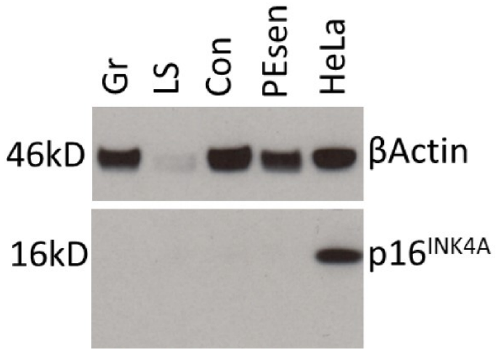

Supplementary Figure S17. p16^{INK4A} protein level in PEsen NHOF-1 and growth arrest controls.

Representative western blot of p16^{INK4A} protein levels in PEsen NHOF-1 cells as well as growing (Gr), serum starved (LS) and confluent (Con) controls, with β -actin loading control. Blots were performed separately on lysates from three independent experiments. White space between rows of bands depicts where the membrane has been cut for incubation with different primary antibodies. The exposure times may be different for each section of membrane. Uncropped images of films used to make this figure can be seen in supplementary figures S18H and S18I.

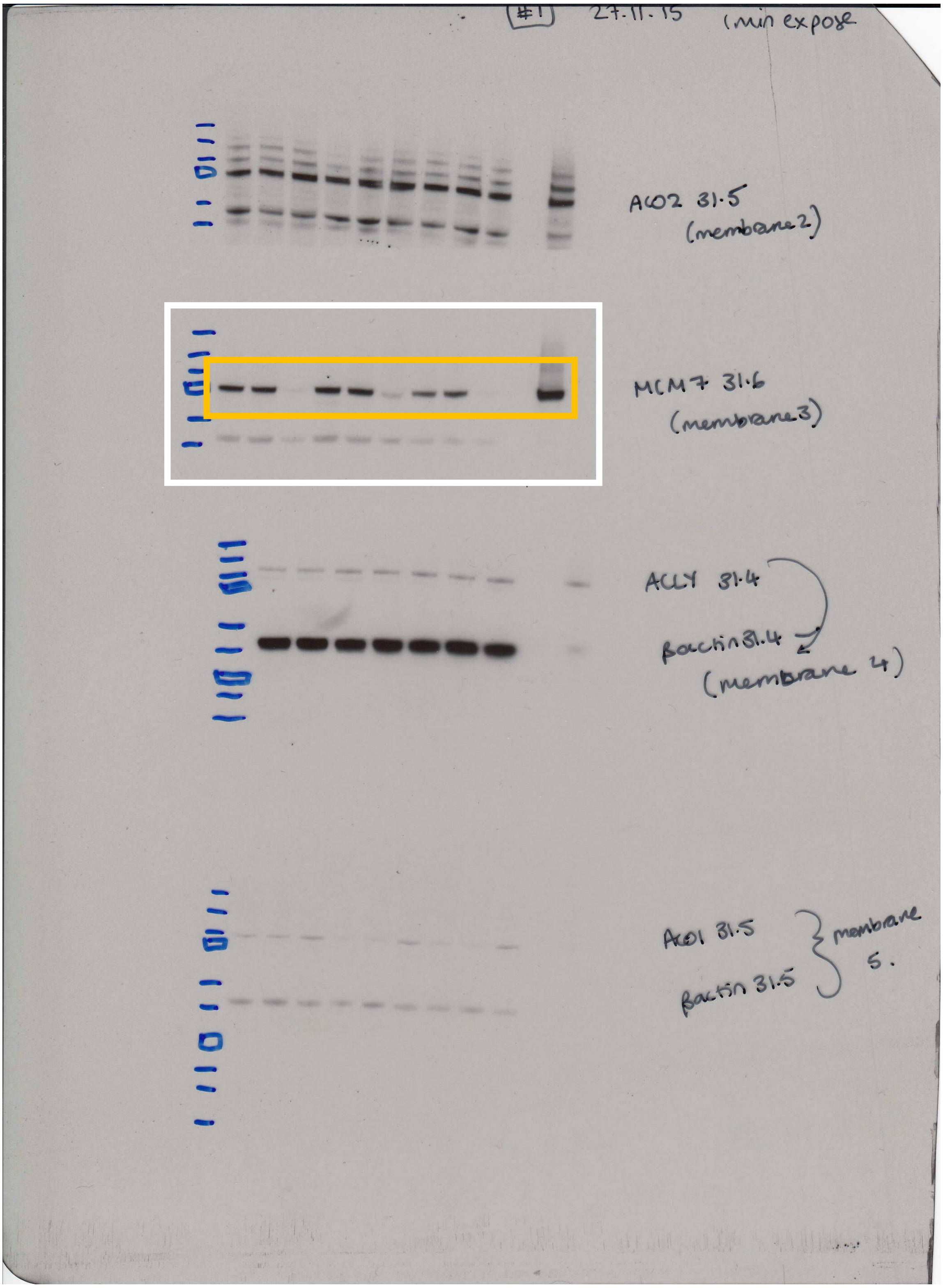

Supplementary figure S18A: white box shows area of membrane and where membrane was cut prior to antibody incubations. Yellow box shows the MCM7 bands that are used in figure S8

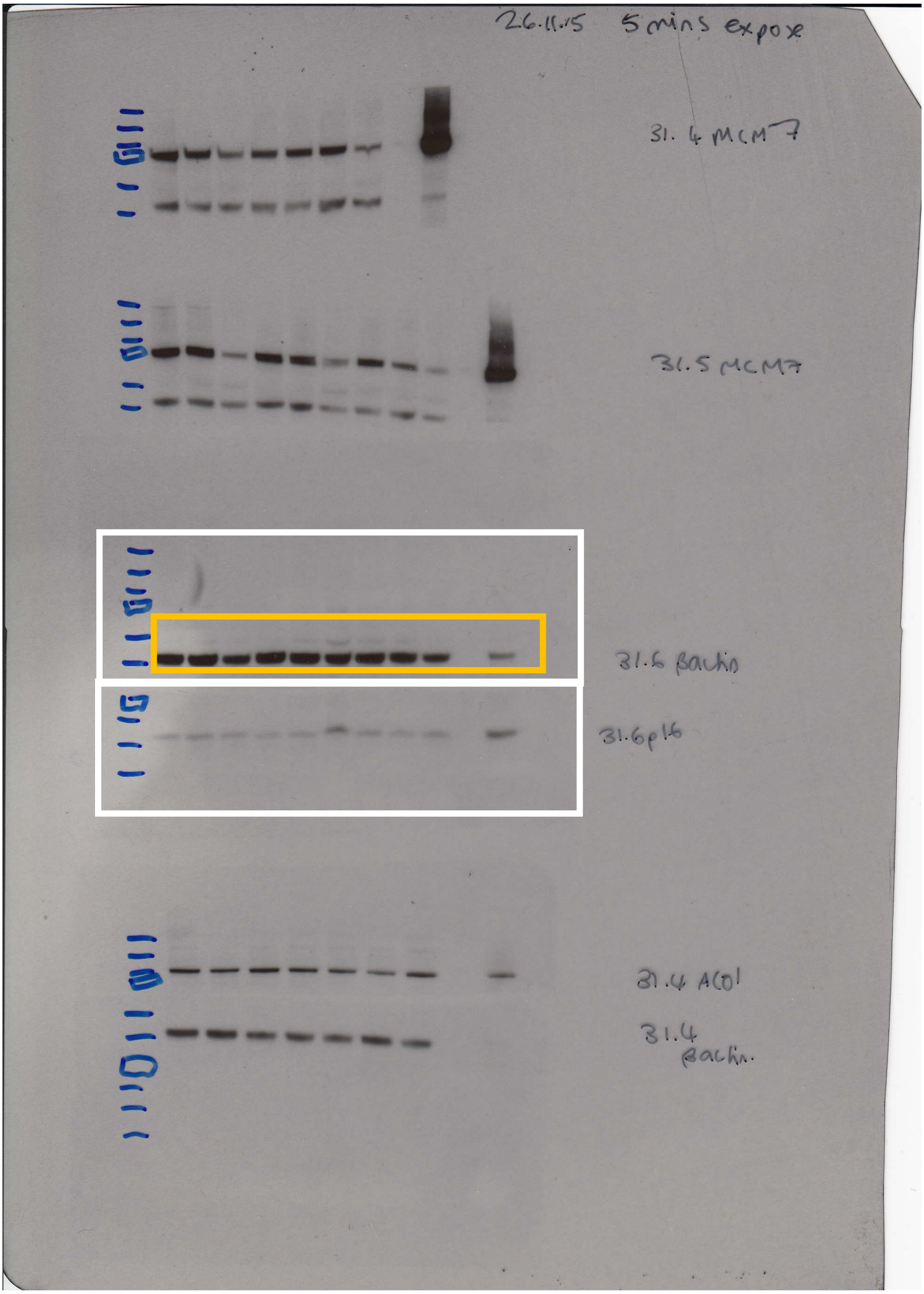

Supplementary figure S18B: white box shows area of membrane and where membrane was cut prior to antibody incubations. Yellow box shows the beta actin bands that are used in figure S8

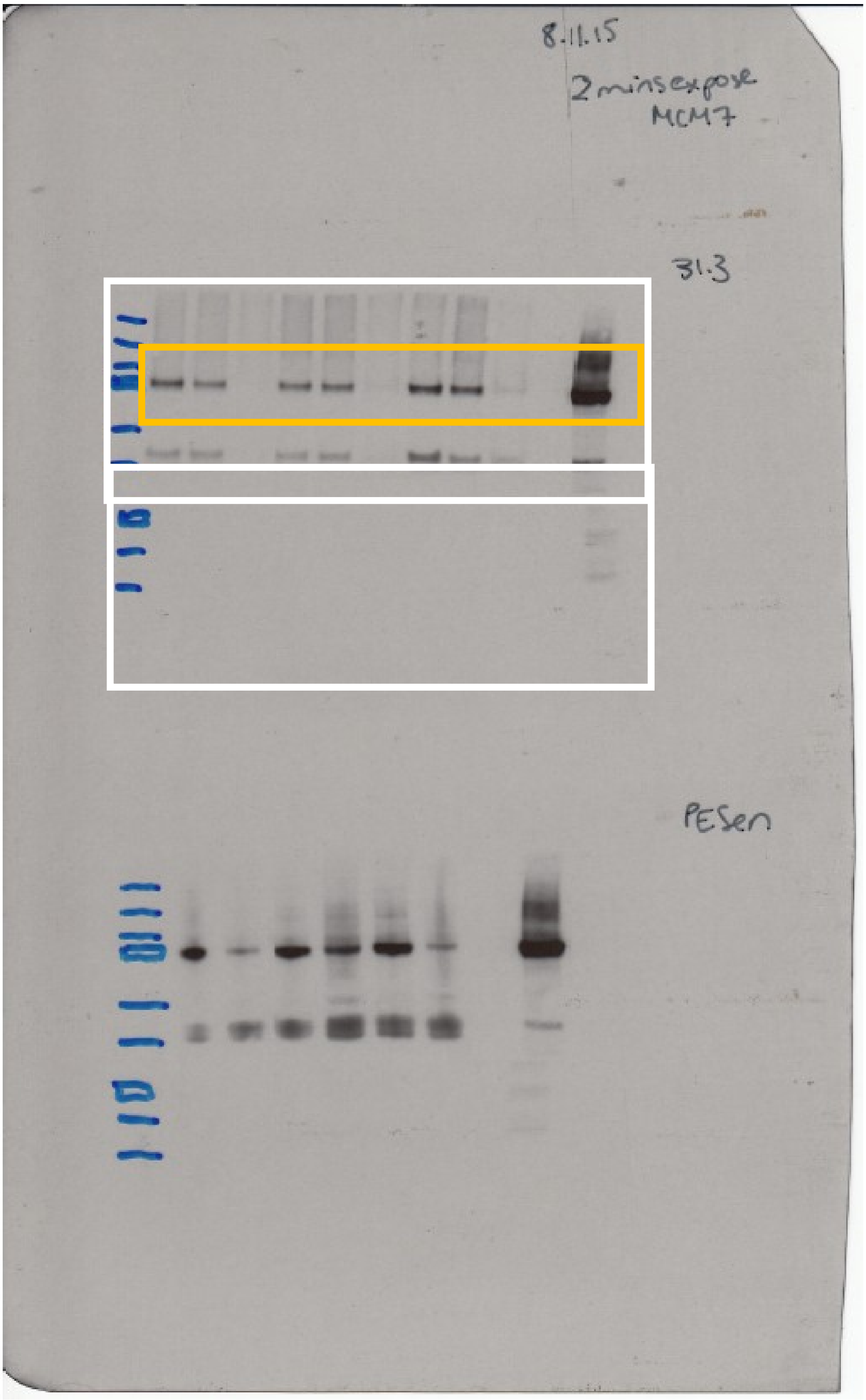

Supplementary figure S18C: white box shows area of membrane and where membrane was cut prior to antibody incubations. Yellow box shows the MCM7 bands that are used in figure S9.

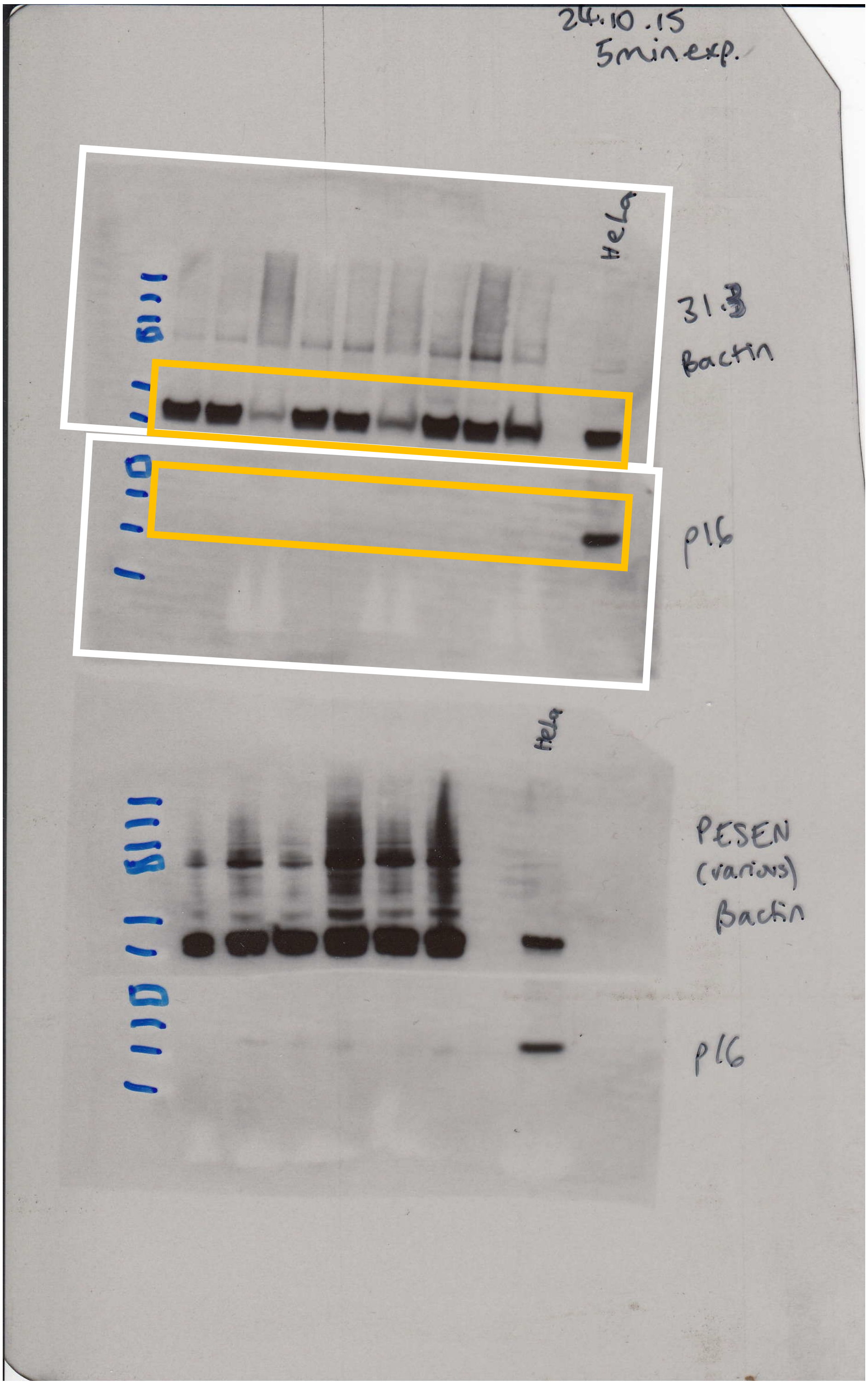

Supplementary figure S18Di: white box shows area of membrane and where membrane was cut prior to antibody incubations. The top yellow box shows the beta actin bands that are used in figure S9 and S13, the bottom yellow box shows the p16^{INK4a} bands that are used in figure S13, after 5 minutes exposure of the film to the membrane.

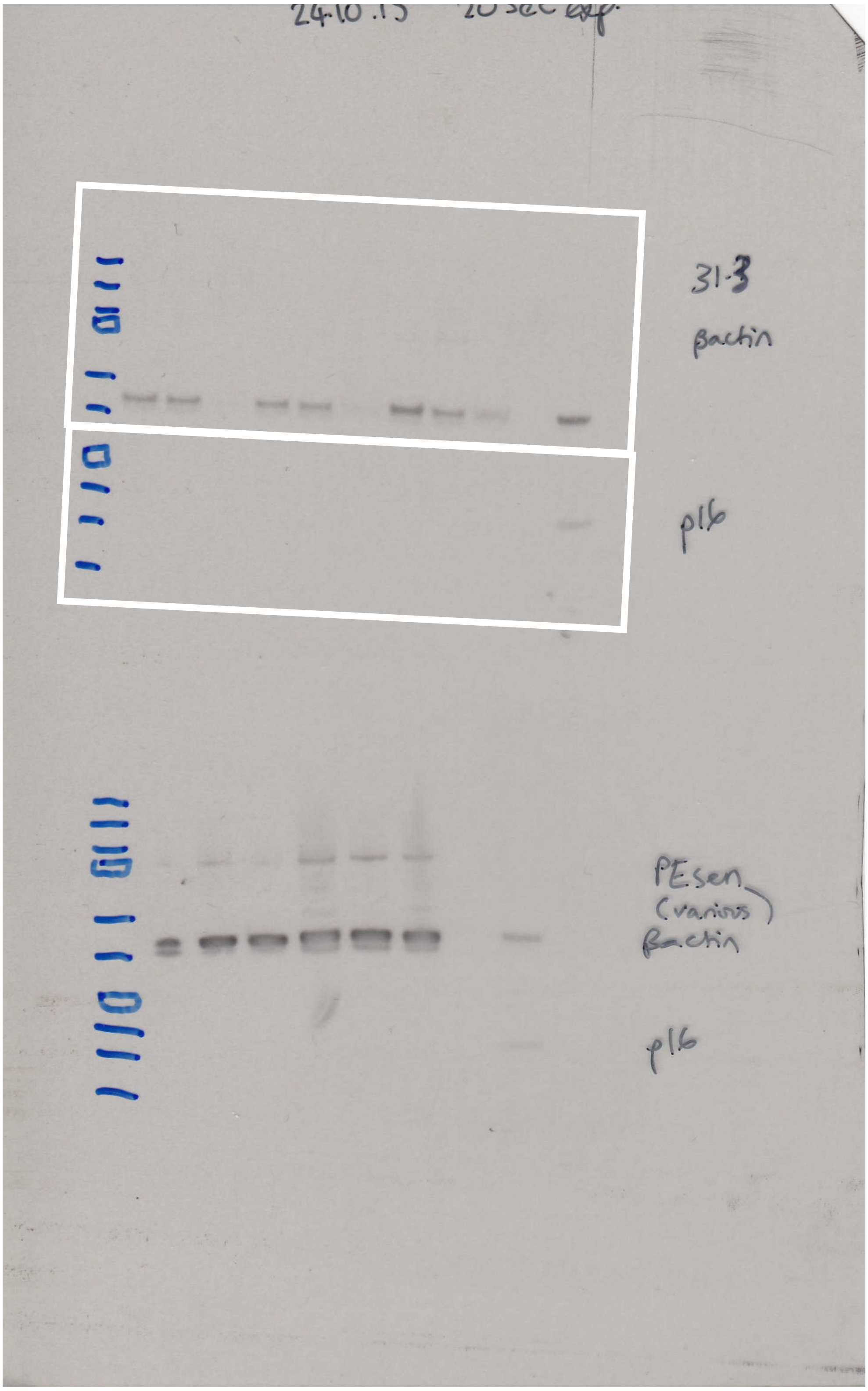

Supplementary figure S18Dii: the same blots shown in SF1a but at an exposure time of 20 seconds.

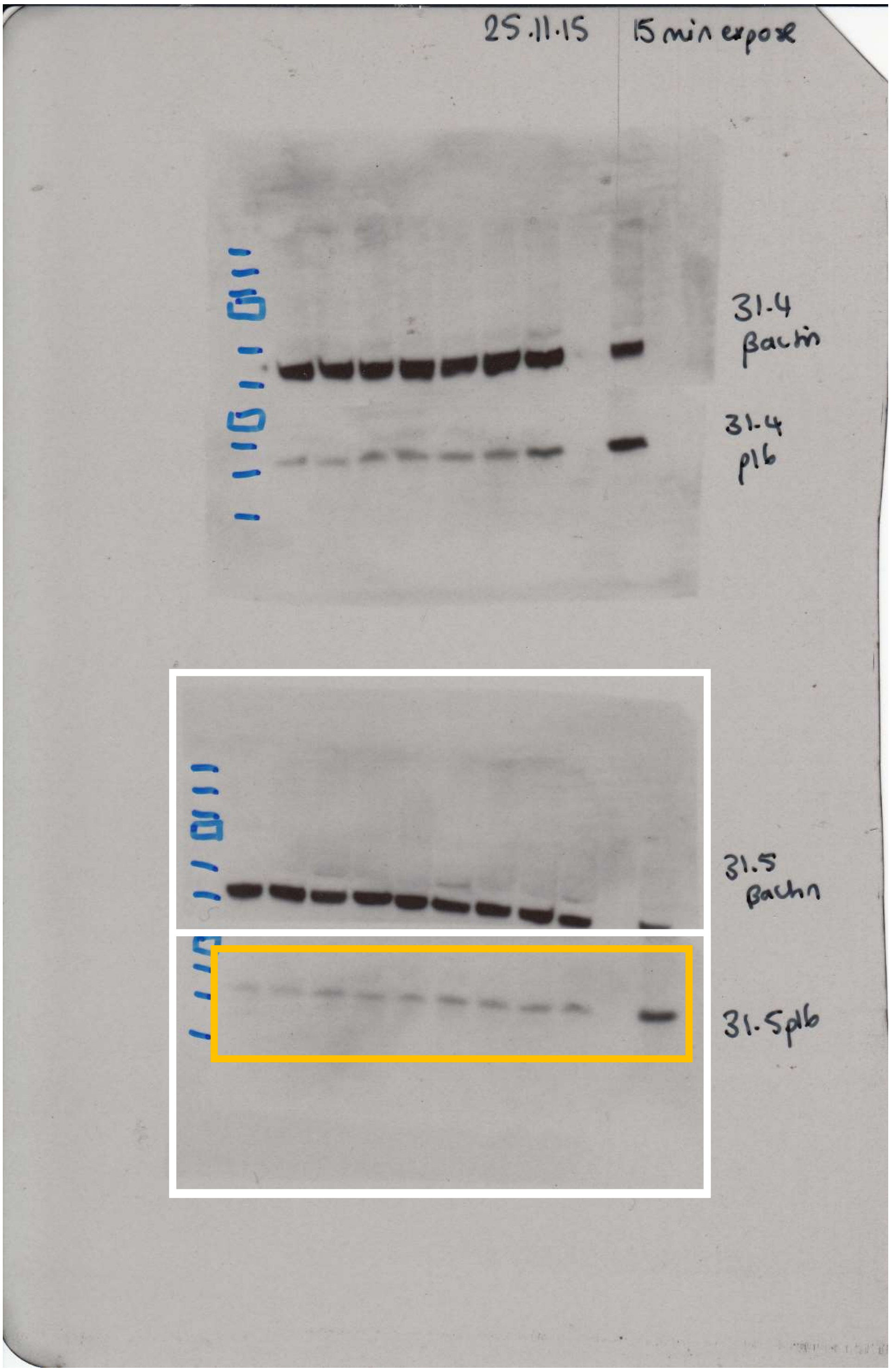

Supplementary figure S18E: white box shows area of membrane and where membrane was cut prior to antibody incubations. Yellow box shows the p16^{INK4a} bands that are used in figure S12

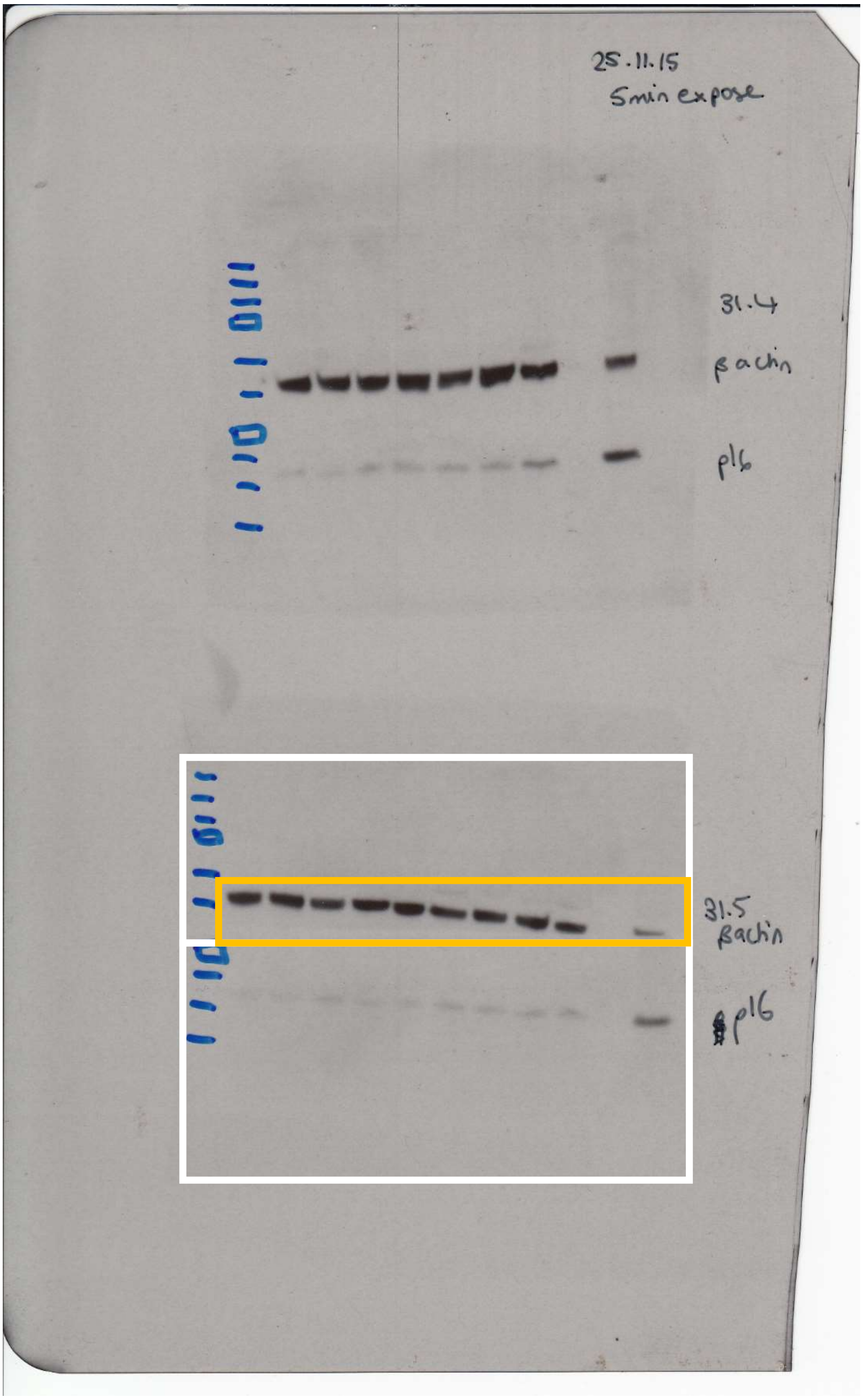

Supplementary figure S18F: white box shows area of membrane and where membrane was cut prior to antibody incubations. Yellow box shows the beta actin bands that are used in figure S12

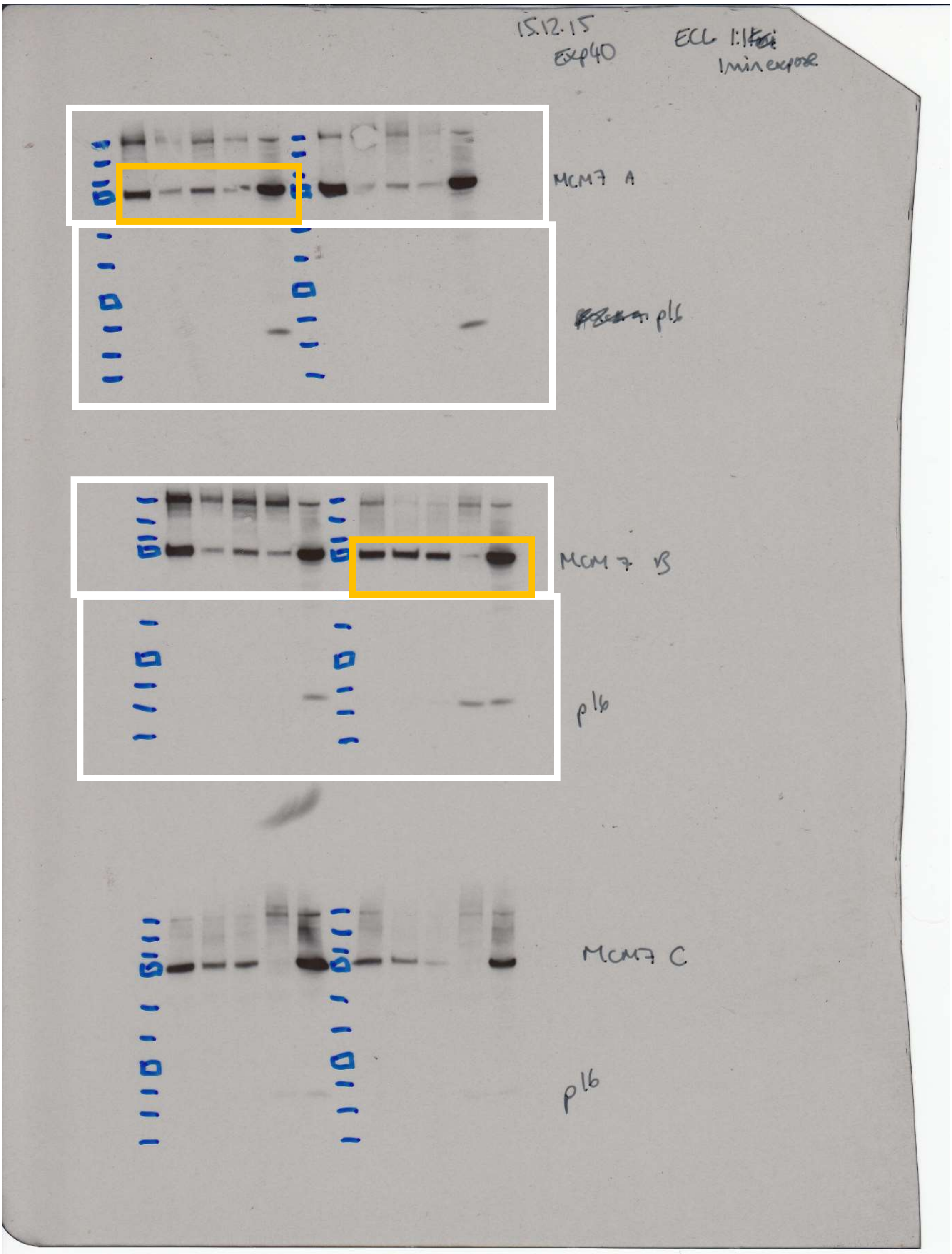

Supplementary figure S18G: white box shows area of membrane and where membrane was cut prior to antibody incubations. Top yellow box shows the MCM7 bands that are used in figure S15B, bottom yellow box shows the MCM7 bands that are used in figure S15A.

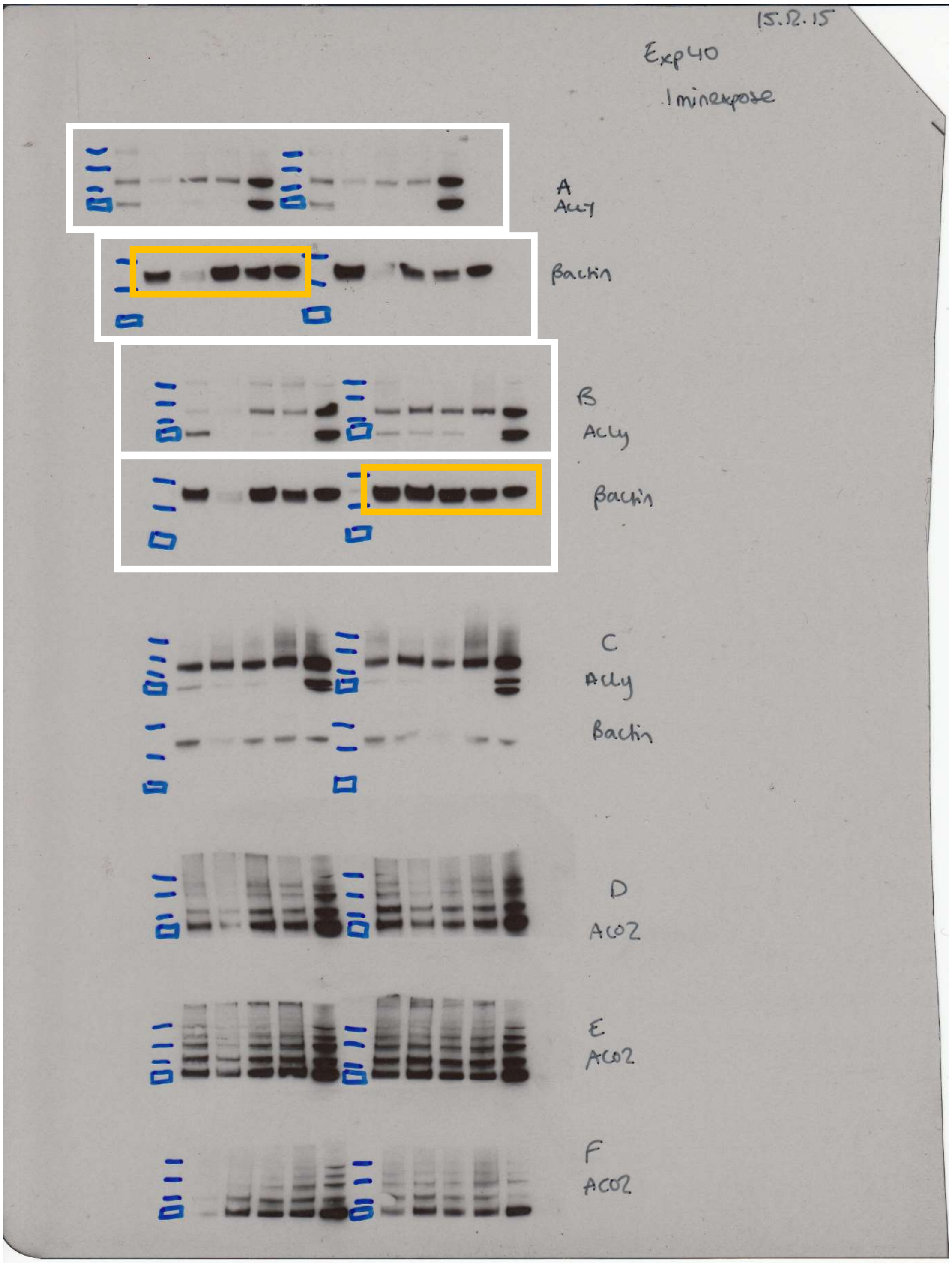

Supplementary figure S18H: white box shows area of membrane and where membrane was cut prior to antibody incubations. Top yellow box shows the beta actin bands that are used in figure S15B, bottom yellow box shows the beta actin bands that are used in Figure S15A.

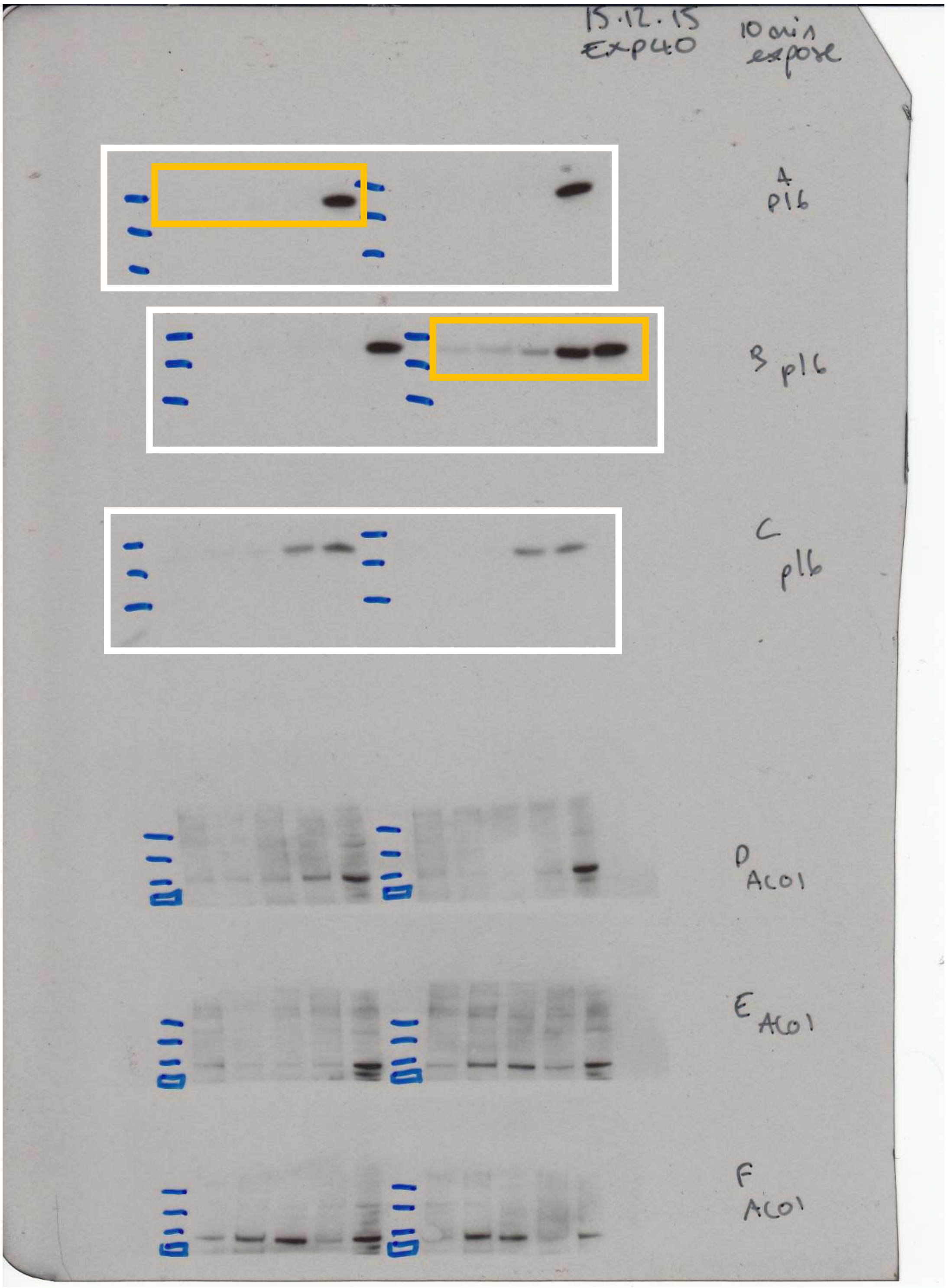

Supplementary figure S18I: white box shows area of membrane and where membrane was cut prior to antibody incubations. Top yellow box shows the p16^{INK4A} bands that are used in figure S16, bottom yellow box shows the p16^{INK4A} bands that are used in Figure S17.

Supplementary Table S1A Metabolites altered relative to unirradiated controls in oral fibroblasts 20 days after the induction of IrrDSBs.

**Metabolite**	**NHOF-1 n =3**	**NHOF-5 n =3**	**Combined n=6**	**Associated with chronological ageing**
**1-stearoylglycerophosphoinositol**	0.1835	0.0276	0.0067	
2-aminoadipate	0.0151	0.0412	0.0008	
**2-hydroxystearate**	0.0073	0.0105	0.0005	**Yes**
3-aminoisobutyrate	0.0715	0.0552	0.0075	
**3-hydroxyisobutyrate**	0.0035	0.0008	1.08E-06	
alpha-lipoate	0.093505	0.110178	0.0334	
beta-hydroxyisovalerate	0.126337	0.136435	0.0224	
caprate (10:0)	0.0428	0.0156	0.0010	**Yes**
caprylate (8:0)	0.0225	0.0254	0.0021	**Yes**
**citrate***	0.3659	0.0755	0.0654	**Yes**
cholate	0.1632	0.1693	0.0277	
**cysteine-glutathione disulfide**	0.0331	0.0475	0.0047	
cytidine	0.1340	0.0729	0.0074	
**dihomo-linoleate (20:2n6)**	0.2340	0.2984	0.0233	**Yes**
eicosenoate (20:1n9 or 11)	0.0021	0.0935	0.0008	**Yes**
erythronate	0.0853	0.0869	0.0287	**Yes**
**gamma-glutamylglutamine**	0.0911	0.0777	0.0163	
**gamma-glutamylleucine**	0.0484	0.5044	0.0392	**Yes**
**gamma-glutamylmethionine**	0.0118	0.2099	0.0050	
**gamma-glutamyltyrosine**	0.0532	0.2010	0.0135	**Yes**
glutathione, oxidized (GSSG)	0.0050	0.0145	2.29E-05	
glycerate	0.0025	0.3654	0.0133	**Yes**

glycochenodeoxycholate	0.1599	0.0977	0.0372	**Yes**
**glycylisoleucine**	**0.0329**	0.1337	**0.0038**	
**glycylleucine**	0.0822	**0.0402**	**0.0066**	
**glycylphenylalanine**	**0.0216**	0.1530	**0.0031**	
**glycylvaline**	**0.0350**	0.0705	**0.0018**	
glycodeoxycholate	0.3072	0.0573	**0.0361**	
isovalerate	0.0792	0.2853	**0.0219**	
malate	**0.0155**	0.4777	**0.0206**	**Yes**
N-acetylphenylalanine	0.0944	0.4069	**0.0494**	
phenol red	0.4385	**0.0409**	**0.0352**	**NA**
phenol sulphate	0.0567	0.1080	**0.0054**	**Yes**
pyridoxal	**0.0221**	**0.0441**	**0.0007**	
**pyridoxate**	**0.0110**	**0.0186**	**0.0001**	**Yes**
**thymidine**	0.0527	0.2277	**0.0232**	
trans-4-hydroxyproline	0.1038	0.0636	**0.0079**	**Yes**
uracil	0.0903	0.2164	**0.0176**	
**urate**	0.1631	0.1272	**0.0182**	**Yes**
uridine	0.268204	0.088424	**0.0218**	**Yes**

Supplementary Table S1B Metabolites altered relative to unirradiated controls in oral fibroblasts 5, 10 and 20 days after the induction of IrrDSBs and their relationship to chronological ageing.

Metabolite	5 Days	10 Days	20 Days	Associated with chronological ageing
13-HODE + 9-HODE	0.5689	0.0336	0.8498	No
**1-stearoylglycerophosphoinositol**	0.0072	0.0030	0.0067	No
2-aminoadipate	0.0002	0.0006	0.0008	No
2-hydroxybutyrate	0.0175	0.1271	0.0672	**Yes**
**2-hydroxystearate**	0.0167	0.0145	0.0005	**Yes**
3-(4-hydroxyphenyl)lactate	0.0221	0.1276	0.5533	**Yes**
3-aminoisobutyrate	0.0061	0.1658	0.0075	No
**3-hydroxyisobutyrate**	1.14E-05	5.45E-05	1.08E-06	No
alpha-lipoate	0.2328	0.0056	0.0334	No
asparagine	0.0336	0.1573	0.1167	**Yes**
beta-hydroxyisovalerate	0.0127	0.0127	0.0224	No
caprate (10:0)	0.2370	0.4467	0.0010	**Yes**
caprylate (8:0)	0.0010	0.0002	0.0021	**Yes**
**citrate***	0.4608	0.1288	0.0654	**Yes**
Cholate	0.4253	0.0947	0.0277	No
**cysteine-glutathione disulfide**	0.0518	0.0042	0.0047	No
Cytidine	0.0048	0.0031	0.0074	No
**dihomo-linoleate (20:2n6)**	0.5784	0.0422	0.0233	**Yes**
eicosenoate (20:1n9 or 11)	0.0763	0.0020	0.0008	**Yes**

erythronate	0.2875	0.7434	**0.0287**	**Yes**
**gamma-glutamylglutamine**	0.0956	**0.0097**	**0.0163**	No
**gamma-glutamylleucine**	0.4173	0.5681	**0.0392**	**Yes**
**gamma-glutamylmethionine**	0.3144	**0.0395**	**0.0050**	No
**gamma-glutamyltyrosine**	0.1935	0.1578	**0.0135**	**Yes**
glutathione, oxidized (GSSG)	**0.0533**	**0.0043**	**2.29E-05**	No
glycerate	0.1850	0.3974	**0.0133**	**Yes**
glycochenodeoxycholate	0.1823	**0.0723**	**0.0372**	**Yes**
**glycylisoleucine**	0.2006	**0.0008**	**0.0038**	No
**glycylleucine**	**0.0601**	**0.0002**	**0.0066**	No
**glycylphenylalanine**	0.5097	**0.0055**	**0.0031**	No
**glycylvaline**	0.1695	**0.0035**	**0.0018**	No
glycodeoxycholate	0.4119	0.5559	**0.0361**	No
isovalerate	**0.0095**	**0.0729**	**0.0219**	No
malate	0.8561	0.3021	**0.0206**	**Yes**
N-acetylphenylalanine	0.9648	0.9009	**0.0494**	No
phenol red	**0.0533**	0.3471	**0.0352**	**NA**
phenol sulphate	**0.0419**	**0.0025**	**0.0054**	**Yes**
pyridoxal	0.1461	**4.11E-06**	**0.0007**	No
**pyridoxate**	**0.0025**	**0.0012**	**0.0001**	**Yes**
**thymidine**	0.0842	**0.0084**	**0.0232**	No
trans-4-hydroxyproline	**0.0201**	**0.0079**	**0.0079**	**Yes**
uracil	**0.0245**	**0.0078**	**0.0176**	No

**urate**	0.2577	0.1419	**0.0182**	**Yes**
**uridine**	**0.0113**	**0.0011**	**0.0218**	**Yes**

The figures in bold were also detected in the PEsen screen (1)

Supplementary Table S2 Novel Metabolites altered in IrrDSBsen fibroblasts compared to growing fibroblasts and their relationship to PEsen.

Pathway	Metabolite	Screen 1 N = 6	Screen 2 N = 9	Combined N = 15	Independent of Cell Cycle Arrest/Quiescence N = 3
Lysine metabolism	2-aminoadipate	**0.0008**	**ND**	**0.0008**	**0.1490**
Pantothenate and CoA metabloism	alpha lipoate	**0.033425**	**0.0069**	**0.0005**	**0.0589**
Isoleucine, leucine and valine (Branch Chain Amino Acid) metabolism	3-hydroxyisobutyrate	**1.08414E-06**	**ND**	**1.08414E-06**	**0.0458**
Alanine and aspartate metabolism	asparagine	**0.1167**	**0.1989**	**0.0436**	**0.0374**
Fatty acid metabolism	isovalerate	**0.0219**	**0.0024**	**0.0009**	**0.0932**
Medium change fatty acid	caprate (10.0)	**0.0010**	**ND**	**0.0010**	**0.2078**
Medium change fatty acid	caprylate (8.0)	**0.0079**	**0.2691**	**0.0021**	**0.0716**
Urea cycle, argenine and proline metabolism	trans-4-hydroxyproline	**0.0079**	**0.2426**	**0.031**	**0.041**
Glutamate metabolism	glutamate	**0.75**	**0.0029**	**0.0127**	**0.055**
Alanine and aspartate metabolism	aspartate	**ND**	**0.0019**	**0.0019**	**0.1568**
Long chain fatty acid	myristoleate (14:1n5)	**ND**	**0.0555**	**0.0555**	**ND**

Values highlighted in red and bold are accumulating in the ESM and differ statistically from the growing and/or confluent controls by the two tailed T test. Figures highlighted in green are becoming depleted significantly from the controls and figures highlighted in pink are showing a trend P > 0.05 but < 0.10.

Supplementary Table S3 Metabolites independently associated with chronological ageing, age-associated disease and cellular senescence

Pathway	Metabolite	PEsen	IrrDSBsen
**Alanine and aspartate metabolism**	**aspartate**	**No**	**Yes**
Creatine metabolism	creatine	No	No
Creatine metabolism	creatinine	No	No
**Glutamate metabolism**	**glutamate**	**Yes[a]**	**Yes**
Glycine, serine threonine metabolism	serine	No	No
**Tryptophan metabolism**	**C-glycosyltryptophan**	**Yes**	**ND**
Urea cycle; arginine, proline, metabolism	citrulline	No	No
Pentose Metabolism	threitol	ND	ND
**Krebs cycle**	**citrate**	**Yes**	**Yes**
Oxidative phosphorylation	phosphate	No	Yes
Carnitine metabolism	octanoylcarnitine	ND	ND
Sterol/steroid	4-androsten-3beta, 17beta-dioldmonosulfateb	ND	ND
Sterol/steroid	17beta-diol disulfate	ND	ND
**Essential fatty acid**	**Eicosapentaenoate (EPA; 20:5n3)**	**Yes**	**ND**
Fatty acid,	3-carboxy-4-methyl-5-	ND	ND

dicarboxylate	propyl-2-<br><br>furanpropanoate (CMPF)		
Long-chain fatty acid	10-heptadecenoate (17:1n7)	Yes	Yes
Long-chain fatty acid	dihomo-linoleate (20:2n6)	Yes	Yes
**Long-chain fatty acid**	**myristoleate (14:1n5)**	**ND**	**Yes[b]**
Sphingolipid	palmitoyl sphingomyelin	No	Yes
**Purine and urate metabolism**	**urate**	**Yes**	**Yes**
Sugar, sugar substitute, starch	erythritol	No	Yes
Xanthine metabolism	1,7-dimethylurate	ND	ND

[a] indicates not significantly different from confluent controls but $P < 0.10$.

[b] indicates not significant from unirradiated controls but $P < 0.10$

Metabolites in red and bold fulfil the criteria of being statistically different in senescent cells from all sets of controls (growing, serum-starved and confluent). Metabolites in pink show a trend and metabolites in green are depleted and hence show the opposite pattern from chronological ageing.

Supplementary Methods

Supplementary method 1:Ki67 and 53BP1 labelling and image acquisition

Adherent cells grown on glass slides were fixed with 4% weight/vol formaldehyde at room temperature for 45 minutes. Cells were permiablised for 20 minutes in PBS containing 1% vol/vol Triton X-100, before blocking in 0.1% weight/vol bovine serum albumin (BSA) in PBS-T (blocking buffer) for 30 minutes at room temperature. Primary antibodies were diluted in blocking buffer (DAKO mouse monoclonal anti-Ki67 clone MIB-1, lot number 20003301, Millepore
mouse kappa monoclonal anti 53BP1 MAb 3802 lot number 2279539, and incubated at room temperature in a humidity chamber for 2 hours.
Following a wash in PBS-T slides were incubated with secondary antibody (Abcam Alexa Fluor 488 goat anti-mouse IgG (H+L) A11001 lot number 1219843) for 1 hour protected from light in the humidity chamber. Following a last wash in PBS-T, coverslips were applied using Vectashield Mounting Medium with DAPI (H-1200 Vector Laboratories).

Images were obtained using the MetaMorph software package (MetaMorph Microscopy Automation & Image Analysis Software, Molecular Devices LLC) on a Leica DM4000B epi-fuorescence upright microscope with QIClick camera containing a Sony IC285 CCD chip for image acquisition. Gain and exposure time were set using young proliferating cells as positive control for Ki67, and senescent cells as a negative control for Ki67 and vice versa as controls for 53BP1. A minimum of 3 12 bit images were taken in different locations within each well, so that a minimum of 100 cells were imaged from different areas of the well.

Supplementary method 2:Ki67 scoring using ImageJ

All scoring was carried out using the open source image analysis software ImageJ and FIJI (Rasband, 1997; Schneider et al., 2012). The process described below has been scripted as a macro, with the help of Dr. Steven West at the University of Oxford, which can be performed using either ImageJ or Fiji, in a semi-supervised manner (the computer runs through the analysis itself, but the images are displayed on the screen for the user to check in case the thresholding encountered any problems, as is sometimes the case with completely negative images. If there were any problems noticed with a particular image, the user has to go back and manually score that image). The DAPI image was auto-thresholded using Otsu's thresholding algorithm (OTSU) and dark background settings. The `fill holes' and `watershed' tools were used to automatically add a 1 pixel thick line to separate any touching nuclei. Nuclei were then defined as regions of interest (ROI) using `analyse particles' options, excluding any objects touching the edges of the image, and including anything over 400 pixels in size to be shown as an outline and added to the ROI manager. The corresponding Alexa 488 image was then opened, auto thresholded in the same way (although importantly no ‘fill holes’ or ‘watershed’ was applied to these images) and the `analyse particles' options were applied to show count masks of objects over 400 pixels in size, that have circularity between 0-1 and exclude any objects on the edge. These measurements were not added to the ROI manager. The ROI manager was then used to show the outlines of the nuclei overlaid on the count masks from the Alexa 488 image, by selecting `show all'. Using the `measure' tool in the ROI manager tool bar produces a table containing information on the size, shape, and integrated density. Objects with 0 recorded for integrated density were recorded as negative for Ki67, objects with a positive value for

integrated density were recorded as positive. The results were exported to an Excel spreadsheet, and the overall score was recorded as number of positive nuclei as a percentage of total nuclei.

Supplementary method 3:53BP1 scoring using ImageJ

As 53BP1 foci are also in the nucleus, first the ROI was defined as described for the analysis of Ki67 staining in supplementary method 2. Then using positive and negative control cells (young proliferating as negative and previously verified senescent cells as positive) a value of noise tolerance was defined in `find maxima' tool such that any foci that would manually be scored as large in the positive control are detected as maxima, but any small foci in the negative control are not picked up. The value for noise tolerance was kept constant for all images, and `find maxima' was used to report a foci as a single point. The ROI were then overlaid and `measure' from the ROI toolbar was used to generate a table of measurements, including integrated density, of each nuclei. The integrated density of one pixel (single point) is 255, so the integrated density of each nucleus was divided by 255 to obtain the number of foci in each nucleus, and the overall scoring reports the percentage of cells that scored positive (i.e. had at least one large foci).

Supplementary References

1. James EL, Michalek RD, Pitiyage GN, de Castro AM, Vignola KS, Jones J, et al. Senescent human fibroblasts show increased glycolysis and redox homeostasis with extracellular metabolomes that overlap with those of irreparable DNA damage, aging, and disease. J Proteome Res. 2015 Apr 03;14(4):1854-71.
